# Resting heart rate causally affects the brain cortical structure: Mendelian randomization study

**DOI:** 10.1093/cercor/bhad536

**Published:** 2024-01-11

**Authors:** Yinsheng Zhong, Jun Li, Yinghui Hong, Shujun Yang, Liying Pei, Xuxiang Chen, Haidong Wu, Tong Wang

**Affiliations:** Department of Emergency, the Eighth Affiliated Hospital of Sun Yat-sen University, Shenzhen, Guangdong 518003, P. R. China; Department of Emergency, the Eighth Affiliated Hospital of Sun Yat-sen University, Shenzhen, Guangdong 518003, P. R. China; Department of Emergency, the Eighth Affiliated Hospital of Sun Yat-sen University, Shenzhen, Guangdong 518003, P. R. China; Department of Emergency, the Eighth Affiliated Hospital of Sun Yat-sen University, Shenzhen, Guangdong 518003, P. R. China; Department of Emergency, the Eighth Affiliated Hospital of Sun Yat-sen University, Shenzhen, Guangdong 518003, P. R. China; Department of Emergency, the Eighth Affiliated Hospital of Sun Yat-sen University, Shenzhen, Guangdong 518003, P. R. China; Department of Emergency, the Eighth Affiliated Hospital of Sun Yat-sen University, Shenzhen, Guangdong 518003, P. R. China; Department of Emergency, the Eighth Affiliated Hospital of Sun Yat-sen University, Shenzhen, Guangdong 518003, P. R. China

**Keywords:** resting heart rate, heart rate variability, cortical structure, Mendelian randomization

## Abstract

Resting heart rate (RHR) has been linked to impaired cortical structure in observational studies. However, the extent to which this association is potentially causal has not been determined. Using genetic data, this study aimed to reveal the causal effect of RHR on brain cortical structure. A Two-Sample Mendelian randomization (MR) analysis was conducted. Sensitivity analyses, weighted median, MR Pleiotropy residual sum and outlier, and MR-Egger regression were conducted to evaluate heterogeneity and pleiotropy. A causal relationship between RHR and cortical structures was identified by MR analysis. On the global scale, elevated RHR was found to decrease global surface area (SA; *P* < 0.0125). On a regional scale, the elevated RHR significantly decreased the SA of pars triangularis without global weighted (*P* = 1.58 × 10^−4^) and the thickness (TH) of the paracentral with global weighted (*P* = 3.56 × 10^−5^), whereas it increased the TH of banks of the superior temporal sulcus in the presence of global weighted (*P* = 1.04 × 10^−4^). MR study provided evidence that RHR might be causally linked to brain cortical structure, which offers a different way to understand the heart–brain axis theory.

## Introduction

Cardiovascular diseases (CVD), such as ischemic heart disease, heart failure, and atherosclerosis, can impair cognitive performance and brain structure (Hajduk et al. 2013; Jefferson et al. 2015; Frey et al. 2021; Vishwanath et al. 2023), which support the presence of a heart–brain axis (Hooghiemstra et al. 2017). Neurovisceral integration modeling also demonstrates a strong relationship between the altered heart rate and brain structure (Thayer and Lane 2000; Thayer et al. 2012). The heart assumes a crucial role in the circulatory system, and the resting heart rate (RHR) is regarded, to a certain extent, as an indicator for maintaining hemodynamic stability. Other studies have shown that not only the hemodynamic balance of the heart–brain axis but also altered heart rate may play a crucial role in maintaining brain structural and functional integrity, as well as maintaining cognitive function (Kisler et al. 2017; Sposato et al. 2017; Matusik et al. 2023).

Elevated RHR is a major modifiable factor in morbidity and mortality worldwide and leads to adverse cardiovascular events and dementia (Aune et al. 2017; Huang et al. 2019; Deng et al. 2022). Prospective and retrospective cohort studies have demonstrated that cognitive decline, dementia, and mood adjustment may be associated with RHR (Thayer et al. 2012; Jennings et al. 2015; Forte et al. 2019). Meanwhile, basal experiments revealed the correlation between increased RHR and activation of the insular cortex (Klein et al. 2021), which triggers anxiety states in mice (Hsueh et al. 2023). Although the correlation between elevated RHR-induced cognitive impairment may be mediated through cortical structures (Goldstein et al. 1998; Haring et al. 2020), the underlying pathophysiological mechanisms between heart rate, cortical structure, and cognitive impairment remain unknown. Therefore, the determination of the specific cortical structures associated with RHR dependence is of clinical implications.

Mendelian randomization (MR), a recently developed analytic method, has been widely used to infer causal associations in which genetic variation is used as a tool for risk factors (Richmond and Davey 2022). Since genetic variants are randomly assigned at the time of conception, MR analysis can theoretically avoid bias from confounding factors in observational studies (Smith and Ebrahim 2003; Davey Smith and Hemani 2014). Some MR studies have reported a causal correlations between RHR variation and other diseases, such as cerebral small vessel disease, diabetes, and atrial fibrillation (Long et al. 2019; Siland et al. 2022; Tian et al. 2023). However, the causal associations between RHR and brain cortical structure have not been demonstrated using MR analysis. We conducted a Two-Sample MR analysis to investigate the associations of RHR on the cerebral cortical structure based on publicly available large-scale population genome-wide association study (GWAS) data.

## Methods

### Study design

**Fig. 1:**
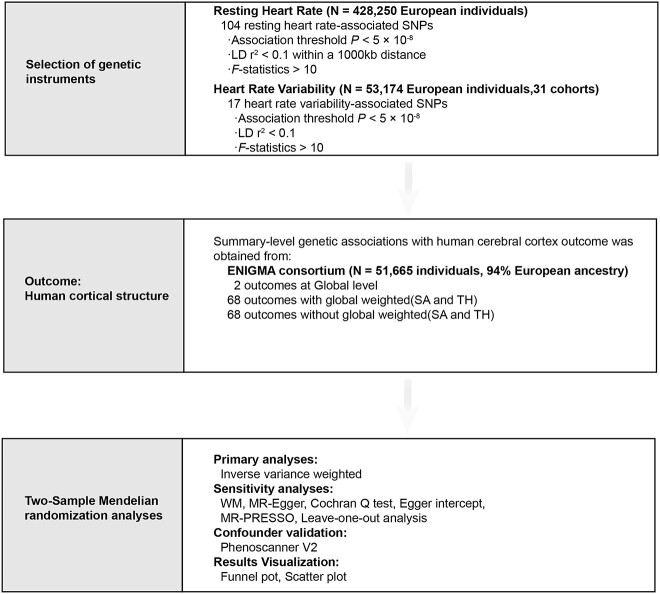
Overview of the Mendelian randomization study. ENIGMA, enhancing NeuroImaging genetics through meta-analysis.

The study overview and the MR analysis procedure is presented in Fig. 1. MR studies satisfy the following assumptions: (i) instrumental variables are related to exposure, (ii) instrumental variables are not associated with confounding factors, and (iii) instrumental variables have direct effect on the outcome only through exposure. Given RHR can be reflected in baseline levels (RHR) and dynamic fluctuations (heart rate variability, HRV) (Monfredi et al. 2014), two sets of instruments, RHR and HRV, were used to perform MR analysis. Cortical structure data were examined by Magnetic Resonance Imaging (MRI) for human cortical surface area (SA) and cortical thickness (TH).

In the MR study, RHR, HRV, and cerebral cortex GWAS were derived from publicly published accessible data (Nolte et al. 2017; Guo et al. 2019; Grasby et al. 2020). Supplementary Table S1 displays the details of data sources. No separate ethical approval was required for this study.

### Data sources for RHR and HRV

The GWAS statistics for RHR were extracted from 428,250 European individuals without beta-adrenergic receptor blockers in the UK Biobank. A total of 437 single-nucleotide polymorphisms (SNPs) were selected at the genome-wide significance level (*P* < 5 × 10^−8^) (Guo et al. 2019). Based on empirical estimates of linkage disequilibrium (LD) between genetic variants, the RHR genome-wide scan was reduced to a subset of independent loci using the PLINK clumping function (*r*^2^ threshold = 0.1, and Window size = 1,000 kb). This parameter has been widely used in previous MR studies (Choi et al. 2019; Cai et al. 2022). The 437 SNPs were matched against the RHR from NHGRI-EBI GWAS Catalog to ensure reliable data (Sollis et al. 2023), and 104 SNPs associated with RHR were retained in the end (Supplementary Table S2). Association analysis adjusted for age, age^2^, sex, genotyping array, and 20 ancestry principal components by the original investigators.

Genetic information for HRV was obtained from a two-stage meta-analysis of GWASs involving 53,174 individuals of European ancestry (Nolte et al. 2017). Based on the 1,000 Genomes database, the LD among SNPs was calculated using the PLINK clustering method, and SNPs in LD were excluded (defined as *r^2^* > 0.1). Further information can be obtained in the original manuscript by Nolte et al. (2017). Finally, it was found that 17 SNPs were significant at the genome-wide level (*P* < 5 × 10^−8^) for HRV (Supplementary Table S3).

### Data source for cerebral cortex SA and TH

Summary GWAS data related to cerebral cortex structure from the ENIGMA consortium (Grasby et al. 2020), which included a genome-wide association meta-analysis of cortical SA and TH measurements from 51,665 individuals worldwide (~94% European ancestry). According to the Desikan–Killiany atlas, the cerebral cortical regions were roughly divided into 34 gyri (Desikan et al. 2006). The MRI measurements were averaged for the same region between both hemispheres. Data with global weighted refer to the global measure as a covariate, and conversely, data without global weighted refer to the global measure not performed as a covariate.

### Statistical analysis

The main statistical analysis method, the random-effects inverse-variance weighted (IVW), was applied to estimate the associations of RHR and HRV with cerebral cortex structure. Several other MR models, including weighted median, MR-Egger, and MR pleiotropy residual sum and outlier (MR-PRESSO), were used as complementary methods. Assuming more than 50% weight from valid instrumental variables, the weighted median method can provide valid MR estimates (Bowden, Davey Smith, et al. 2016). MR-Egger regression can detect horizontal pleiotropy by its intercept (*P* < 0.05 was considered as the presence of pleiotropy) and generate estimates after correcting for pleiotropic effects although the method consumes statistical power (Bowden et al. 2015; Burgess and Thompson 2017). The MR-PRESSO method can detect outliers and generate causal estimates after removing outlying SNPs (Verbanck et al. 2018). The STEIGER test was used to determine for the presence of reverse causality (Hemani et al. 2017). In order to satisfy the first assumption, we calculated the *F*-statistic for each SNP to assess the statistical strength. We further used funnel pot and leave-one-out analyses to detect the presence of pleiotropy and assess the robustness of the MR estimates. In addition, for SNPs associated with potential risk factors (e.g. neuropsychiatric disorders, obesity, hyperlipidemia, hypertension, smoking, and alcohol consumption), we verified them using PhenoScanner (www.phenoscanner.medschl.cam.ac.uk) (Kamat et al. 2019) and removed the confounding SNPs at the genome-wide significance level. To take into account multiple testing for different regions, the associations with *P*-values below 1.84 × 10^−4^ (*α* = 0.05/272 outcomes) were regarded significant associations after Bonferroni correction and associations with *P-*values below 0.05 but above 1.84 × 10^−4^ were deemed as nominally significant. A significant two-sided threshold was 0.0125 (*α* = 0.05/4 outcomes) for the global level test. All analyses were performed using the TwoSampleMR (0.5.6) and MR-PRESSO (1.0) packages in R version 4.2.2.

## Results

The *F*-statistic for each genetic variant of the exposed instrument was greater than 10 and was considered to have no weak instrumental variables (Pierce et al. 2011) (Specific details are in Supplementary Tables S2 and S3). Data were harmonized to omit SNPs with directionally inconsistent alleles and palindromic SNPs were removed from the analysis. All data successfully passed the Steiger direction test, providing conclusive evidence of the absence of reverse causality (Supplementary Table S5). Based on the genetic instrument selection procedure, we finally identified 81 indexed SNPs on RHR and 16 indexed SNPs on HRV, of which there were two overlaps (rs236349, rs6123471).

**Fig. 2:**
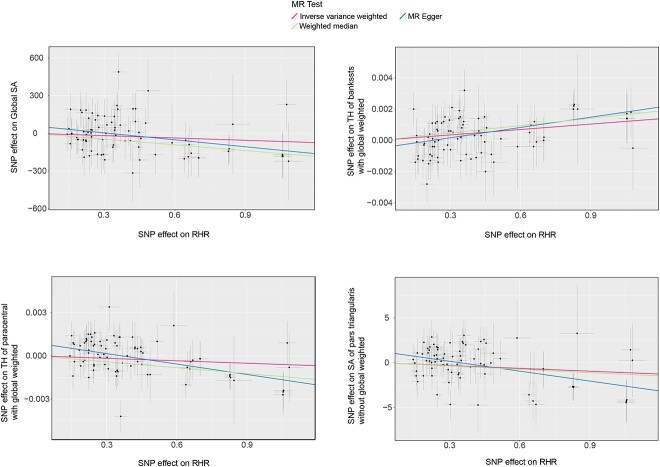
Scatterplots for the significant MR association (*P* < 1.84 × 10^−4^) between RHR and cortical structure.

We performed MR analysis of global and regional functional gyrus using the selected genetic instruments (Table 1). At the global level, elevated RHR was found to reduce global SA (β_SA_ = −185.20 mm^2^, 95% CI: −322.62 to −47.78 mm^2^, *P* < 0.0125), no associations were detected on global TH (β_TH_ = 7.65 × 10^−5^ mm, 95% CI: −5.23 × 10^−4^ to 6.76 × 10^−4^ mm, *P* = 0.802). Pleiotropy was observed with MR-Egger intercept *P* < 0.05 and MR-Egger as the primary outcome (Fig. 2). The *P*-value for Cochran *Q* is >0.05, suggesting that there is no heterogeneity. Genetically predicted HRV had no causality with global SA and TH (β_SA_ = 563.33 mm^2^, 95% CI: −462.95 to 1589.61 mm^2^, *P* = 0.282; β_TH_ = −0.004 mm, 95% CI: −0.014 to 0.006 mm, *P* = 0.468).

**Table 1:** Significant and nominal significant MR estimates from RHR and HRV on genetically predicted cortical structure.

**Exposure**		**Outcome**	**β (95% CI)**	**IVW-derived *P*-value**	**Heterogeneity (Q statistic, *P*)**	**Pleiotropy (Intercept, *P*)**	**Distortion Test (*P*)**
Global	RHR	Total SA^**^	−185.20 mm^2^ (−322.62 to −47.78 mm^2^)	0.01^†^	81.63, 0.525	59.42,0.046	
Regions with global weighted	RHR	TH of banks of the superior temporal sulcus^***^	0.0012 mm (0.0006–0.0017 mm)	1.04 × 10^−4^	72.25, 0.719	−0.0005, 0.054	
		TH of paracentral^***^	−0.0024 mm (−0.0035 to −0.0013 mm)	3.56 × 10^–5†^	81.24, 0.409	−0.0009, 2.36 × 10^−4^	
		SA of entorhinal	0.33 mm^2^ (0.03–0.62 mm^2^)	0.031	77.91, 0.545	−0.211, 0.105	
		SA of fusiform	1.75 mm^2^ (0.35–3.15 mm^2^)	0.014	114.76, 0.007	0.143, 0.817	
		SA of inferior temporal	4.14 mm^2^ (1.43–6.85 mm^2^)	0.004^†^	79.2, 0.473	−1.332, 0.024	
		SA of lingual	1.50 mm^2^ (0.05–2.94 mm^2^)	0.043	83.03, 0.386	−0.491, 0.439	
		SA of paracentral	0.79 mm^2^ (0.04–1.53 mm^2^)	0.039	92.77, 0.156	0.177, 0.589	
		SA of pars triangularis	−1.95 mm^2^ (−3.50 to −0.39 mm^2^)	0.016^†^	69.5, 0.769	0.752, 0.026	
		SA of precentral	3.60 mm^2^ (0.42–6.78 mm^2^)	0.029^†^	73.74, 0.646	−1.381, 0.046	
		SA of rostral middle frontal	−3.26 mm^2^ (−5.37to −1.14 mm^2^)	0.003	101.68, 0.051	0.316, 0.734	
		SA of superior temporal	−1.67 mm^2^ (−2.92 to −0.43 mm^2^)	0.008	101.16, 0.055	0.358, 0.512	
		SA of temporal pole	−0.30 mm^2^ (−0.52 to −0.07 mm^2^)	0.009	66.53, 0.859	0.058, 0.554	
		TH of caudal anterior cingulate	0.001 mm (3.23 × 10^−5^ to 0.0019 mm)	0.043	50.28, 0.996	−0.0002, 0.67	
		TH of fusiform	0.0014 mm (0.0003–0.0024 mm)	0.017^†^	96.9, 0.084	−0.0005, 0.023	
		TH of inferior parietal	0.0005 mm (0.0001–0.0009 mm)	0.008	84.94, 0.332	−0.0002, 0.319	
		TH of lateral occipital	0.0016 mm (0.0007–0.0026 mm)	0.001^†^	91.85, 0.153	−0.0005, 0.009	
		TH of medial orbitofrontal	−0.0007 mm (−0.0014 to −0.0001 mm)	0.026	75.57, 0.619	−0.00007, 0.8	
		TH of pars opercularis	−0.0006 mm (−0.0011 to −0.0003 mm)	0.02^‡^	NA	NA	0.54
		TH of pars triangularis	0.0018 mm (0.0007–0.0029 mm)	0.002^†^	101.62, 0.044	−0.0006, 0.01	
		TH of postcentral	−0.0007 mm (−0.0012 to −0.0003 mm)	0.002	102.69, 0.045	0.0001, 0.544	
		TH of posterior cingulate	0.0008 mm (0.0002–0.0014 mm)	0.01	88.18, 0.249	−0.0002, 0.46	
		TH of precentral	−0.0006 mm (−0.001 to −2.16 × 10^−5^ mm)	0.045^‡^	NA	NA	0.83
		TH of superior frontal	0.0006 mm (0.0001–0.001 mm)	0.01	97.42, 0.090	−0.00005, 0.787	
		TH of temporal pole	0.0047 mm (0.002–0.0074 mm)	0.001^†^	84.27, 0.322	−0.0014, 0.016	
Regions without global weighted	RHR	SA of pars triangularis^***^	−3.69 mm^2^ (−5.51 to −1.89 mm^2^)	1.58 × 10^–4†^	78.37, 0.499	1.266, 0.002	
		SA of caudal anterior cingulate	−0.71 mm^2^ (−1.31 to −0.10 mm^2^)	0.023	90.02, 0.208	0.255, 0.338	
		SA of insula	−1.633 mm^2^ (−2.812 to −0.455 mm^2^)	0.008^‡^	NA	NA	0.53
		SA of isthmus cingulate	−0.95 mm^2^ (−1.70 to −0.2mm^2^)	0.015^‡^	NA	NA	0.59
		SA of middle temporal	−2.33 mm^2^ (−4.16 to −0.49 mm^2^)	0.013	83.16, 0.382	1.469, 0.065	
		SA of pars opercularis	−2.17 mm^2^ (−4.27 to −0.08 mm^2^)	0.045^†^	82.72, 0.365	0.967, 0.034	
		SA of pars orbitalis	−1.17 mm^2^ (−2.02 to −0.31 mm^2^)	0.009^†^	98.14, 0.071	0.365, 0.049	
		SA of posterior cingulate	−2.30 mm^2^ (−3.79 to −0.82 mm^2^)	0.003^†^	72.64, 0.68	0.740, 0.022	
		SA of rostral middle frontal	−12.01 mm^2^ (−18.65 to −5.37 mm^2^)	0.001^†^	57.44, 0.968	3.009, 0.037	
		SA of superior temporal	−2.90 mm^2^ (−5.11 to −0.68 mm^2^)	0.01	114.02, 0.007	1.624, 0.092	
		SA of supramarginal	−3.65 mm^2^ (−6.22 to −1.07 mm^2^)	0.007^‡^			0.82
		SA of temporal pole	−0.42 mm^2^ (−0.69 to −0.16 mm^2^)	0.002	91.83, 0.172	0.189, 0.098	
		SA of transverse temporal	−0.39 mm^2^ (−0.71 to −0.07 mm^2^)	0.016	96.43, 0.102	−0.105, 0.452	
		TH of banks of the superior temporal sulcus	0.0024 mm (0.0009–0.004 mm)	0.003^†^	61.94, 0.921	−0.0007, 0.049	
		TH of lateral occipital	0.0007 mm (0.0001–0.0014 mm)	0.024^‡^	NA	NA	0.79
		TH of paracentral	−0.0022 mm (−0.0039 to −0.0006 mm)	0.01^†^	104.23, 0.176	0.0007, 0.037	
		TH of pars triangularis	0.0021 mm (0.0005–0.0037 mm)	0.011^†^	112.58, 0.008	−0.0008, 0.026	
		TH of temporal pole	0.0049 mm (0.002–0.0078 mm)	0.002^†^	65.06, 0.87	−0.0016, 0.01	
Regions with global weighted	HRV	SA of temporal pole	3.85 mm^2^ (0.46–7.25 mm^2^)	0.026	7.9, 0.928	0.028, 0.861	
		TH of caudal anterior cingulate	−0.02 mm (−0.03 to −0.003 mm)	0.014	13.41, 0.57	0.0007, 0.305	
		TH of superior temporal	0.01 mm (4.24′10–4 to 0.02 mm)	0.039	15.03, 0.449	0.0004, 0.297	
		TH of supramarginal	−0.01 mm (−0.01 to −9.86′10–5 mm)	0.046	13.92, 0.531	0.0001, 0.66	
Regions without global weighted	HRV	SA of temporal pole	5.95 mm^2^ (2.22–9.67 mm^2^)	0.002	8.74, 0.891	−0.035, 0.842	
		SA of lingual	28.27 mm^2^ (1.41–55.13 mm^2^)	0.039	12.69, 0.626	0.697, 0.589	
		TH of caudal anterior cingulate	−0.02 mm (−0.04 to −0.01 mm)	0.002	15.79, 0.396	0.00006, 0.938	
		TH of posterior cingulate	−0.02 mm (−0.03 to −0.01 mm)	0.007^‡^	NA	NA	0.73
		TH of superior frontal	−0.01 mm (−0.03 to −1.9 × 10^−4^ mm)	0.047	21.34, 0.126	−0.0004, 0.48	

^**^ *P* < 0.0125,^***^P < 1.84 × 10^−4^(Bonferroni correction);

^†^MR estimates derived from MR-Egger method;

^‡^MR estimates (corrected) derived from MR-PRESSO method;

Heterogeneity and pleiotropy *P*-value < 0.05 is significant. CI, Confidence interval; IVW, Inverse-variance weighted; RHR, resting heart rate; HRV, heart rate variability; SA, cortical surface area; TH, cortical thickness.

**Fig. 3:**
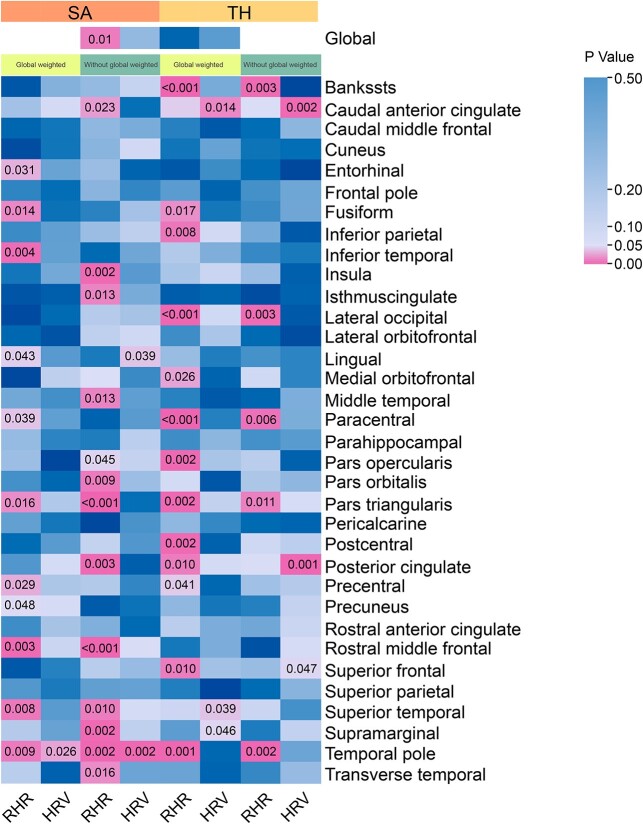
Summary of associations of genetically predicted RHR and HRV with brain cortical structure. Numbers in the boxes represent the *P*-values of every MR analysis. The association with a *P*-value < 0.05, but Bonferroni-adjusted *P*-values > 1.84 × 10^−4^, was considered nominal significant, and adjusted *P*-values < 1.84 × 10^−4^ were deemed significant (regional scale). Bankssts, banks of the superior temporal sulcus.

At the regional functional gyrus level, the elevated RHR significantly increases the TH of banks of the superior temporal sulcus (Bankssts, β_TH_ = 0.001 mm, 95% CI: 0.0006–0.0017 mm, *P* = 1.04 × 10^−4^) and decreases the TH of the paracentral in the presence of global weighted (β_TH_ = −0.002 mm, 95% CI: −0.003 to 0.002 mm, *P* = 3.56 × 10^−5^). We found that elevated RHR was able to significantly decrease the SA of pars triangularis without global weighted (β_SA_ = −3.39 mm^2^, 95% CI: −5.51 to −1.87 mm^2^, *P* = 1.58 × 10^−4^). No heterogeneity was detected above all and pleiotropy was present only in paracentral or pars triangularis. Meanwhile, HRV and RHR nominally affect gyri, including entorhinal, inferior temporal, lingual, precentral, rostral middle frontal, superior temporal, temporal pole, caudal anterior cingulate, fusiform, inferior parietal, lateral occipital, medial orbitofrontal, postcentral, posterior cingulate, superior frontal, pars opercularis, supramarginal, and transverse temporal gyrus (Supplementary Table S6 and Fig. 3). All significant and nominal significant estimates were visualized and analyzed using leave-one-out and funnel plots (Supplementary Figs. S1–S14).

To avoid other risk factors that could affect the causal estimates, we examined SNPs one by one in the Phenoscanner. We found that SNPs within RHR exclusively exhibited potential risk factors, while none were identified within HRV. After removing SNPs associated with potential risk factors (Supplementary Table S4), MR analysis was performed again. The estimates remain consistent with the initial results (Global: β_SA_ = −72.94 mm^2^, 95% CI: −142.39 to −3.49 mm^2^, *P* = 0.039; Banks of the superior temporal sulcus: β_TH_ = 0.001 mm, 95% CI: 0.0006–0.0018 mm, *P* = 1.17 × 10^−4^; Paracentral:β_TH_ = −0.003 mm, 95% CI: −0.004 to −0.002 mm, *P* = 2.63 × 10^−5^; Pars triangularis: β_SA_ = −3.63 mm^2^, 95% CI: −5.49 to −1.77 mm^2^, *P* = 2.79 × 10^−4^), suggesting that RHR has robust estimates of causal effects for the TH of banks of the superior temporal sulcus and paracentral with global weighted and the SA of pars triangularis without global weighted.

## Discussion

In our MR study, we provided the first causal evidence between heart rate variations (HRV and RHR) and cerebral cortex structure using large-scale MR analysis that could provide reliable evidence for the causal relationship. We found the atrophy of the Global SA, the SA of pars triangularis, and the TH of paracentral are associated with elevated RHR. Meanwhile, RHR increased the TH of Bankssts. It partially explains the pathophysiological underpinnings of how changes in heart rate interact with brain function to support the notion that there is a heart–brain axis.

RHR and HRV are considered to be signatures of autonomic nervous system activity. Observational study shows higher RHR and lower HRV associated with an increased risk of cognitive decline and dementia (da Silva et al. 2018; Imahori et al. 2022). The Atherosclerosis Risk in Communities Study with 20-year follow-up describes that RHR is prospectively associated with cognitive decline in middle-aged adults without stroke and atrial fibrillation (Wang et al. 2019). Further studies revealed subcortical lesions, asymptomatic infarcts, and white matter high signal volume in older adults with elevated RHR (Nakanishi et al. 2018). There is abundant evidence that an elevated RHR is associated with the development of cardiovascular disease events (Aune et al. 2015; Tadic et al. 2018). In a cohort study including 2,147 elderly participants without CVD, elevated RHR was associated with an increased risk of dementia and a faster rate of cognitive decline (Imahori et al. 2022). Our findings suggest that elevated RHR is associated with a reduction in the SA of the overall cortex and the unweighted SA of the Pars triangularis. Research indicates that the reduction in the SA of the Pars triangularis is involved in physiological symptoms (Estevez-Lopez et al. 2023), the processing of sentences with unstated iterative meaning (Lai et al. 2023), and the onset of schizophrenia (Guardiola-Ripoll et al. 2023). Meanwhile, our evidence also suggests that RHR has the potential to influence the TH of the Bankssts and the paracentral. The Bankssts gray matter volume is positively correlated with potential depression (Bashford-Largo et al. 2023). A reduction in the TH of the paracentral lobule cortex in individuals at high risk for mental health issues indicates psychological vulnerability (Sasabayashi et al. 2021). Excitingly, Hsueh et al. (2023) observed in a murine experiment that a transient increase in RHR (660–900 bpm/min) strongly activates the posterior insular cortex (posterior insula) and the brainstem. Although the experiment had a short duration, preventing the observation of long-term effects on cortical structures. This suggests that RHR may be able to serve as a noninvasive treatment for mental disorders by altering the cortical structure of the brain. Generally, higher RHR indicated poorer brain function. However, we found estimates in our results that differed from logical expectations. The genetically predicted higher RHR was positively correlated with the TH of Bankssts. Similarly, we observed a positive correlation between the other functional gyrus and elevated RHR. Given the complexity of the cerebral cortex, there may be some compensatory mechanism for the effects of RHR on functional gyrus (cerebral hypertrophy or edema). Nevertheless, elevated RHR indeed altered the cortical structure, which was consistent with previous studies (Deng et al. 2022).

Functional neuroimaging studies have shown the correlation between brain structure and HRV. In a study including 30 healthy young individuals, HRV was observed to be positively associated with the TH of different hemispheric functional gyrus. The only negative correlation between HRV and isthmus cingulate cortex was observed in the left hemisphere (Winkelmann et al. 2017). A meta-analysis showed that the TH of the lateral orbitofrontal cortex in the left hemisphere was positively correlated with HRV in age-adjusted pairs of noninterest cortex thicknesses (Yoo et al. 2018). We performed MR analysis considering the limitations of cross-sectional studies. Our results indicate a suggestive association between HRV and regional brain cortical structures. In a 5-week HRV biofeedback intervention trial, increased HRV may improve inhibitory control (Nashiro et al. 2023). Our evidence provides a theoretical biological basis for this study.

In addition, higher RHR and lower HRV are also associated with an increased risk of various CVD, including stroke and cerebrovascular lesions (Aune et al. 2017; Pavlovic 2021), which are also risk factors for the development of dementia (Cannon et al. 2017; Wolters et al. 2018). We examined SNPs in Phenoscanner to test whether our results were interfered with by potential risk factors. In addition, some of the results of our MR analysis have pleiotropy where IVW estimates are biased. In this situation, the MR-Egger method should be used, as it allows SNP pleiotropy to adjust the IVW analysis (Bowden, Del Greco, et al. 2016), provided that the beta direction remains consistent for all MR methods (Cheng et al. 2022).

There are several strengths in the present study. Firstly, to our knowledge, the MR study is the first to investigate the causal relationship between heart rate variation and cerebral cortical structure, which minimized confounding factors and avoided reverse causation. What is more, we used two sets of genetic instruments to represent heart rate variation from different aspects to ensure robust MR analysis, thus increasing the chance of identifying significant estimates. The reporting of this study conforms to the Strengthening the Reporting of Observational Studies in Epidemiology guidelines (Supplementary Table S7). The limitations of our study also need to be considered carefully. The predominant portion of individuals in the summary data we employed exhibited European ancestry. The constraints imposed by this population composition may curtail the generalizability of our findings. Limited overlap may exist between the cohorts used in the exposure and outcome MR analyses, which is unavoidable due to the use of summary-level data. To avoid the weak instrument bias in MR analyses brought by potential overlapped datasets, the *F*-statistic > 10 for this analysis indicated that the bias introduced by sample overlap should be minimal. We have just illustrated the causal inference between heart rate variation and structural changes in the cerebral cortex, but the mechanisms need to be investigated further. Future research is needed to further explore the underlying mechanisms by which heart rate affects cerebral cortex structure, which may provide new treatment targets for patients with neuropsychiatric disorders.

## Conclusions

This is the first MR study to confirm the association between RHR variation and cerebral cortex structure. We found evidence of causal associations that elevated RHR decreased global SA, the SA of pars triangularis, the TH of paracentral, and increased the TH of Bankssts, which may point to additional connection in the heart–brain axis. To understand the process behind it, more investigation is needed. These results may inform decisions about potential benefits and risks for patients with neuropsychiatric disorders.

## Supplementary Material

Supplementary_Materials_bhad536

## Data Availability

All data generated or analyzed during this study are included in this published article.
