# Supplementary material for: Resting heart rate causally affects the brain cortical structure: Mendelian randomization study
Source: Cereb Cortex. 2024 Jan 11;34(2):bhad536. doi: 10.1093/cercor/bhad536 (PMC10839837; doi:10.1093/cercor/bhad536)

**Supplemental Material**

**Resting heart rate causally affects the brain cortical structure: Mendelian randomization study**

Yinsheng Zhong^#^, Jun Li^#^, Yinghui Hong^#^, Shujun Yang, Liying Pei, Xuxiang Chen, Haidong Wu, Tong Wang^1^*

Department of Emergency, the Eighth Affiliated Hospital of Sun Yat-sen University, Shenzhen, Guangdong, 518003, P. R. China

# Equal contributors

*Correspondence: tongwang316@163.com

**SUPPLEMENTAL MATERIAL**

[Supplementary Table S1 Information of Summary Statistics Used in MR Study 1](#_Toc144910441)

[Supplementary Table S2 Characteristics of the single-nucleotide polymorphisms associated with resting heart rate 2](#_Toc144910442)

[Supplementary Table S3 Characteristics of the single-nucleotide polymorphisms associated with heart rate variability 5](#_Toc144910443)

[Supplementary Table S4 Related risk traits of the single-nucleotide polymorphisms associated with RHR/HRV from PhenoScanner V2 6](#_Toc144910444)

[Supplementary Table S5 The steiger test for the association between exposure and outcome. 7](#_Toc144910445)

[Supplementary Table S6 Significant and nominal significant all MR estimates from RHR and HRV on genetically predicted cortical structure. 10](#_Toc144910446)

[Supplementary Table S7 STROBE-MR checklist of recommended items to address in reports of Mendelian randomization studies 24](#_Toc144910447)

[Supplementary Fig.1 MR estimates of significant results from RHR and HRV on cortical SA and TH. 29](#_Toc144910448)

[Supplementary Fig.2 MR estimates of significant results from RHR and HRV on cortical SA and TH after removal of risk SNPs in phenoscanner 31](#_Toc144910449)

[Supplementary Fig.3 Scatter plots of nominal significant estimates from genetically predicted RHR with global weighted on the cortical structure. 32](#_Toc144910450)

[Supplementary Fig.4 Scatter plots of nominal significant estimates from genetically predicted RHR without global weighted on the cortical structure. 34](#_Toc144910451)

[Supplementary Fig.5 Scatter plots of nominal significant estimates from genetically predicted HRV with global weighted on the cortical structure. 35](#_Toc144910452)

[Supplementary Fig.6 Scatter plots of nominal significant estimates from genetically predicted HRV without global weighted on the cortical structure 36](#_Toc144910453)

[Supplementary Fig.7 Leave-one-out plots of nominal significant estimates from genetically predicted RHR with global weighted on the cortical structure 37](#_Toc144910454)

[Supplementary Fig.8 Leave-one-out plots of nominal significant estimates from genetically predicted RHR without global weighted on the cortical structure 38](#_Toc144910455)

[Supplementary Fig.9 Leave-one-out plots of nominal significant estimates from genetically predicted HRV with global weighted on the cortical structure 39](#_Toc144910456)

[Supplementary Fig.10 Leave-one-out plots of nominal significant estimates from genetically predicted HRV without global weighted on the cortical structure 40](#_Toc144910457)

[Supplementary Fig.11 Funnel plots of nominal significant estimates from genetically predicted RHR with global weighted on the cortical structure 41](#_Toc144910458)

[Supplementary Fig.12 Funnel plots of nominal significant estimates from genetically predicted RHR without global weighted on the cortical structure. 42](#_Toc144910459)

[Supplementary Fig.13 Funnel plots of nominal significant estimates from genetically predicted HRV with global weighted on the cortical structure. (a) SA of the temporal pole; (b) TH of the caudal anterior cingulate; (c) TH of the superior temporal; (d) TH of the supramarginal 43](#_Toc144910460)

[Supplementary Fig.14 Funnel plots of nominal significant estimates from genetically predicted HRV without global weighted on the cortical structure 44](#_Toc144910461)

Supplementary Table S1 Information of Summary Statistics Used in MR Study

| **Trait** | **Consortium** | **Ancestry** | **Sample size** | **PMID** |
| --- | --- | --- | --- | --- |
| Resting heart rate | UK Biobank study | European | 428,250 | 31648709 |
| Heart rate variability | Meta-analysis | European | 53,174 | 28613276 |
| Cerebral cortex | ENIGMA | 94% European | 51,665 | 32193296 |

ENIGMA, Enhancing NeuroImaging Genetics through Meta Analysis

Supplementary Table S2 Characteristics of the single-nucleotide polymorphisms associated with resting heart rate

| **SNP** | **EA** | | **OA** | **EAF** | **BETA** | **SE** | **P value** | **F** |
| --- | --- | --- | --- | --- | --- | --- | --- | --- |
| rs9970334 | T | | G | 0.551228 | -0.22336 | 0.023213 | 9.3E-23 | 93 |
| rs182770070 | A | | T | 0.988495 | -0.77733 | 0.107144 | 3.0E-13 | 53 |
| rs272564 | A | | C | 0.716545 | -0.43324 | 0.025443 | 1.6E-67 | 290 |
| rs10789207 | T | | C | 0.786421 | 0.339392 | 0.027772 | 4.7E-34 | 149 |
| rs2152735 | G | | A | 0.672628 | 0.272813 | 0.024361 | 5.1E-30 | 125 |
| rs1171563 | T | | G | 0.771212 | 0.143828 | 0.02706 | 2.1E-08 | 28 |
| rs35118522 | T | | C | 0.894861 | -0.6567 | 0.037019 | 2.2E-71 | 315 |
| rs41317993 | G | | A | 0.895237 | -0.66917 | 0.037142 | 1.8E-73 | 325 |
| rs2745959 | T | | C | 0.667102 | 0.4324 | 0.024238 | 2.2E-73 | 318 |
| rs2745967 | G | | A | 0.372538 | -0.44748 | 0.023435 | 6.6E-83 | 365 |
| rs11454451 | C | | CT | 0.743284 | -0.1734 | 0.026222 | 1.0E-11 | 44 |
| rs846111 | G | | C | 0.72752 | -0.23766 | 0.025486 | 1.5E-21 | 87 |
| rs1260326 | T | | C | 0.395414 | 0.293619 | 0.023199 | 2.9E-37 | 160 |
| rs12713404 | G | | T | 0.386704 | 0.222335 | 0.023553 | 9.1E-21 | 89 |
| rs62144050 | T | | C | 0.714035 | -0.2114 | 0.025115 | 8.3E-18 | 71 |
| rs17494056 | A | | C | 0.72613 | 0.156683 | 0.025514 | 7.3E-10 | 38 |
| rs564190295 | G | | GCCGCC  GCCCCC | 0.849335 | 0.18809 | 0.032596 | 3.6E-09 | 33 |
| rs151041685 | G | T | | 0.911836 | -1.07348 | 0.039963 | 3.8E-162 | 722 |
| rs10497529 | G | A | | 0.962546 | -1.08175 | 0.059673 | 2.0E-75 | 329 |
| rs62172372 | A | G | | 0.802552 | -0.36106 | 0.028831 | 1.8E-37 | 157 |
| rs907683 | G | T | | 0.427149 | 0.345055 | 0.023472 | 7.2E-50 | 216 |
| rs4608502 | T | C | | 0.329039 | -0.24639 | 0.024196 | 8.9E-25 | 104 |
| rs13002735 | A | C | | 0.761295 | 0.337085 | 0.026679 | 9.9E-37 | 160 |
| rs41312411 | C | G | | 0.848671 | 0.31917 | 0.031986 | 1.8E-24 | 100 |
| rs6599255 | A | C | | 0.417868 | -0.22411 | 0.023078 | 3.1E-23 | 94 |
| rs3749237 | G | A | | 0.676945 | -0.30263 | 0.024288 | 8.7E-37 | 155 |
| rs2358740 | G | T | | 0.673163 | 0.23189 | 0.024251 | 2.0E-22 | 91 |
| rs1483890 | A | G | | 0.699879 | -0.23473 | 0.024891 | 9.7E-22 | 89 |
| rs11920570 | G | A | | 0.731309 | -0.26326 | 0.025653 | 1.3E-24 | 105 |
| rs7612445 | G | T | | 0.811487 | 0.389064 | 0.029218 | 1.7E-41 | 177 |
| rs12501032 | C | G | | 0.686504 | -0.33098 | 0.024685 | 4.5E-42 | 180 |
| rs6845865 | T | C | | 0.844034 | 0.368647 | 0.031384 | 3.1E-32 | 138 |
| rs13165531 | A | T | | 0.412938 | 0.173874 | 0.023097 | 1.9E-13 | 57 |
| rs9326726 | G | A | | 0.189086 | -0.18351 | 0.029109 | 2.5E-11 | 40 |
| rs1468333 | T | C | | 0.627324 | 0.20608 | 0.023565 | 4.1E-19 | 76 |
| rs4868243 | G | A | | 0.837769 | 0.345934 | 0.03103 | 4.0E-28 | 124 |
| rs2744375 | A | T | | 0.843119 | -0.27347 | 0.031442 | 1.3E-18 | 76 |
| rs236349 | A | G | | 0.338815 | -0.27776 | 0.024122 | 1.9E-31 | 133 |
| rs3951016 | T | A | | 0.531953 | -0.51997 | 0.022879 | 3.5E-117 | 517 |
| rs281868 | G | A | | 0.501951 | 0.476126 | 0.022743 | 1.2E-100 | 438 |
| rs10457327 | G | C | | 0.945585 | -0.90175 | 0.050165 | 4.1E-75 | 323 |
| rs11154022 | A | G | | 0.309588 | -0.40439 | 0.024563 | 1.5E-60 | 271 |
| rs3792943 | C | T | | 0.630331 | -0.38111 | 0.02582 | 1.8E-50 | 218 |
| rs9320841 | A | G | | 0.901555 | -1.05763 | 0.038168 | 1.7E-172 | 768 |
| rs9398652 | C | A | | 0.901584 | -1.05487 | 0.038163 | 9.5E-172 | 764 |
| rs1320761 | C | T | | 0.902456 | -1.05575 | 0.038328 | 7.9E-171 | 759 |
| rs58437978 | T | C | | 0.49272 | 0.207636 | 0.023007 | 3.7E-19 | 81 |
| rs180239 | G | C | | 0.34585 | 0.351086 | 0.023917 | 4.9E-49 | 215 |
| rs140367586 | T | G | | 0.984299 | 0.761765 | 0.100763 | 7.0E-14 | 57 |
| rs314370 | T | C | | 0.811758 | -0.64562 | 0.029091 | 2.8E-112 | 493 |
| rs12666989 | G | C | | 0.817154 | -0.66084 | 0.029376 | 2.8E-115 | 506 |
| rs17881696 | G | A | | 0.816747 | -0.6605 | 0.02936 | 2.3E-115 | 506 |
| rs41748 | T | G | | 0.547622 | 0.224483 | 0.022934 | 1.3E-22 | 96 |
| rs11563648 | G | C | | 0.274595 | 0.199421 | 0.025538 | 9.2E-15 | 61 |
| rs73158705 | A | G | | 0.841172 | -0.4113 | 0.031242 | 3.3E-40 | 173 |
| rs4731790 | T | A | | 0.461232 | 0.251674 | 0.022856 | 8.9E-28 | 121 |
| rs56233017 | G | A | | 0.954363 | 0.588102 | 0.055606 | 5.0E-27 | 112 |
| rs34965012 | G | GT | | 0.571933 | 0.135684 | 0.023129 | 3.9E-09 | 34 |
| rs684573 | A | C | | 0.755378 | 0.150871 | 0.026523 | 9.0E-09 | 32 |
| rs748802 | G | A | | 0.668588 | 0.243995 | 0.024578 | 7.5E-24 | 99 |
| rs10820614 | G | C | | 0.799807 | 0.159389 | 0.028486 | 1.6E-08 | 31 |
| rs10739663 | A | G | | 0.547901 | 0.307746 | 0.022886 | 8.8E-41 | 181 |
| rs12576326 | A | G | | 0.657566 | -0.24989 | 0.023998 | 4.3E-26 | 108 |
| rs174536 | A | C | | 0.652032 | -0.35459 | 0.023928 | 1.8E-51 | 220 |
| rs174547 | T | C | | 0.655734 | -0.35772 | 0.023987 | 5.5E-52 | 222 |
| rs12364753 | G | A | | 0.693404 | 0.15764 | 0.02484 | 1.5E-10 | 40 |
| rs75190942 | C | A | | 0.91055 | 0.450979 | 0.039873 | 8.4E-31 | 128 |
| rs74964634 | C | T | | 0.929329 | -0.31216 | 0.045445 | 8.8E-13 | 47 |
| rs2283274 | G | C | | 0.825236 | 0.534559 | 0.030262 | 5.6E-70 | 312 |
| rs10841486 | T | C | | 0.779745 | 0.301868 | 0.027519 | 3.2E-28 | 120 |
| rs4963772 | G | A | | 0.848874 | 0.701243 | 0.031762 | 1.3E-111 | 487 |
| rs17287293 | A | G | | 0.849528 | 0.700001 | 0.031767 | 2.8E-111 | 486 |
| rs1050288 | C | T | | 0.661653 | 0.244335 | 0.024228 | 1.1E-24 | 102 |
| rs1343676 | T | C | | 0.496301 | 0.446737 | 0.022819 | 3.2E-88 | 383 |
| rs1994135 | T | C | | 0.516547 | -0.45581 | 0.022839 | 4.8E-91 | 398 |
| rs10880689 | A | G | | 0.593761 | -0.2466 | 0.02318 | 1.9E-27 | 113 |
| rs826838 | C | T | | 0.456474 | 0.212354 | 0.022903 | 1.1E-21 | 86 |
| rs867400 | T | C | | 0.424598 | -0.27186 | 0.023078 | 1.0E-32 | 139 |
| rs12579753 | C | T | | 0.770163 | 0.248897 | 0.027094 | 9.7E-20 | 84 |
| rs12889267 | A | G | | 0.832753 | -0.51704 | 0.030466 | 3.2E-67 | 288 |
| rs365990 | A | G | | 0.629992 | -0.82674 | 0.023575 | 1.0E-274 | 1230 |
| rs422068 | T | C | | 0.640758 | -0.83056 | 0.023759 | 5.9E-273 | 1222 |
| rs452036 | G | A | | 0.641723 | -0.829 | 0.023777 | 1.9E-271 | 1216 |
| rs223116 | A | G | | 0.257548 | 0.343076 | 0.026004 | 5.7E-40 | 174 |
| rs17180489 | G | C | | 0.853775 | 0.584241 | 0.03222 | 2.3E-74 | 329 |
| rs1549118 | C | T | | 0.72088 | -0.16167 | 0.025476 | 9.7E-10 | 40 |
| rs17201923 | A | G | | 0.714259 | 0.422319 | 0.025235 | 1.4E-65 | 280 |
| rs4900069 | A | C | | 0.372094 | -0.1898 | 0.023585 | 1.0E-15 | 65 |
| rs7173389 | A | T | | 0.84172 | 0.580692 | 0.031233 | 2.1E-79 | 346 |
| rs1592560 | A | C | | 0.400901 | 0.187584 | 0.023725 | 1.1E-15 | 63 |
| rs3915499 | G | A | | 0.682821 | -0.34945 | 0.024558 | 1.6E-44 | 202 |
| rs7194801 | T | C | | 0.426947 | 0.331886 | 0.023107 | 6.0E-48 | 206 |
| rs79121763 | C | T | | 0.909501 | 0.421292 | 0.040092 | 4.3E-26 | 110 |
| rs12941356 | A | G | | 0.410282 | 0.213725 | 0.023328 | 1.4E-20 | 84 |
| rs117159291 | A | C | | 0.949965 | -0.48672 | 0.052831 | 9.5E-21 | 85 |
| rs11083258 | A | C | | 0.827765 | 0.288718 | 0.030206 | 2.3E-21 | 91 |
| rs11081761 | G | A | | 0.859545 | 0.215253 | 0.032912 | 3.5E-11 | 43 |
| rs61735998 | G | T | | 0.974893 | 0.845469 | 0.073034 | 6.0E-31 | 134 |
| rs16974196 | G | A | | 0.68381 | -0.16338 | 0.024618 | 1.3E-11 | 44 |
| rs12721051 | C | G | | 0.811661 | 0.182339 | 0.029209 | 1.3E-10 | 39 |
| rs6123471 | T | C | | 0.532727 | 0.639644 | 0.022868 | 1.4E-177 | 782 |
| rs17265513 | T | C | | 0.801382 | -0.20051 | 0.028603 | 2.9E-12 | 49 |
| rs2283847 | C | T | | 0.446182 | 0.237698 | 0.023568 | 8.3E-24 | 102 |
| rs2076028 | G | A | | 0.706837 | 0.2969 | 0.025193 | 4.9E-33 | 139 |

SNP, single nucleotide polymorphism; EA, effect allele; OA, other allele; EAF, effect allele frequency; SE, standard error; F, F-statistics.

The summary data is cited from Guo Y, Chung W, Zhu Z, Shan Z, Li J, Liu S, Liang L (2019) J Am Coll Cardiol 74:2162-2174. https://doi.org/10.1016/j.jacc.2019.08.1055

Supplementary Table S3 Characteristics of the single-nucleotide polymorphisms associated with heart rate variability

| **SNP** | **EA** | **OA** | **EAF** | **BETA** | **SE** | **P value** | **F** |
| --- | --- | --- | --- | --- | --- | --- | --- |
| rs10842383 | C | T | 0.87 | -0.124 | 0.012958 | 1.2E-25 | 91 |
| rs12974440 | A | G | 0.07 | -0.243631 | 0.019003 | 1.9E-41 | 165 |
| rs1351682 | G | A | 0.44 | -0.073237 | 0.009215 | 5.7E-15 | 66 |
| rs12974991 | A | G | 0.08 | -0.11701 | 0.008202 | 4.6E-46 | 210 |
| rs180238 | C | T | 0.33 | -0.034439 | 0.004273 | 8.0E-16 | 72 |
| rs1812835 | A | C | 0.42 | -0.02527 | 0.004064 | 5.2E-10 | 39 |
| rs2052015 | T | C | 0.17 | -0.036107 | 0.005752 | 3.6E-10 | 36 |
| rs236349 | G | A | 0.66 | -0.035488 | 0.004264 | 9.1E-17 | 77 |
| rs6123471 | T | C | 0.53 | -0.023783 | 0.00418 | 1.3E-08 | 36 |
| rs7980799 | A | C | 0.39 | -0.038742 | 0.004202 | 3.2E-20 | 95 |
| rs12980262 | A | G | 0.08 | -0.060229 | 0.006044 | 2.3E-23 | 100 |
| rs1384598 | T | A | 0.43 | -0.022636 | 0.003154 | 7.4E-13 | 59 |
| rs2529471 | C | A | 0.43 | -0.02116 | 0.003002 | 1.9E-12 | 49 |
| rs2680344 | A | G | 0.78 | -0.024063 | 0.003657 | 4.9E-11 | 36 |
| rs36423 | T | G | 0.13 | -0.032977 | 0.00458 | 6.3E-13 | 44 |
| rs4262 | C | T | 0.39 | -0.02771 | 0.003294 | 4.3E-17 | 87 |
| rs4899412 | T | C | 0.25 | -0.025882 | 0.003548 | 3.1E-13 | 42 |

SNP, single nucleotide polymorphism; EA, effect allele; OA, other allele; EAF, effect allele frequency; SE, standard error; F, F-statistics.

The summary data is cited from Nolte IM, Munoz ML, Tragante V, Amare AT, Jansen R, Vaez A et al (2017) Nat Commun 8:15805. https://doi.org/10.1038/ncomms15805

Supplementary Table S4 Related risk traits of the single-nucleotide polymorphisms associated with RHR/HRV from PhenoScanner V2

| **Exposure** | **SNP** | **Pos(h19)** | **A1** | **A2** | **Trait** | **Type** | **PMID** | **Beta** | **P** | **N** |
| --- | --- | --- | --- | --- | --- | --- | --- | --- | --- | --- |
| RHR | rs1260326 | chr2:27730940 | C | T | Triglyceride levels | Diseases and traits | 28334899 | -0.115 | 4.00E-253 | - |
|  | rs2358740 | chr3:53455569 | G | T | Schizophrenia | Diseases and traits | 28991256 | 0.05235 | 7.00E-08 | - |
|  | rs3749237 | chr3:49770032 | G | A | Whole body fat mass | Diseases and traits | UKBB | -0.0193 | 2.25E-14 | 330762 |
|  | rs9326726 | chr5:107747447 | A | G | Body mass index | Diseases and traits | UKBB | 0.02284 | 9.61E-14 | 336107 |
|  | rs12721051 | chr19:45422160 | C | G | Medication for cholesterol, blood pressure or diabetes | Diseases and traits | UKBB | -0.028 | 9.31E-48 | 154702 |
|  | rs17265513 | chr20:39832628 | C | T | Body fat percentage | Diseases and traits | UKBB | 0.01399 | 1.85E-09 | 331117 |
|  | rs174536 | chr11:61551927 | A | C | Triglycerides | Diseases and traits | 24097068 | -0.0522 | 1.23E-26 | 91013 |
|  | rs174547 | chr11:61570783 | C | T | Total cholesterol | Diseases and traits | 24097068 | -0.0472 | 1.35E-35 | 184184 |
|  | rs4900069 | chr14:91583373 | A | C | Body mass index | Diseases and traits | UKBB | -0.0113 | 5.31E-06 | 336107 |
|  | rs56233017 | chr8:144981488 | A | G | Whole body fat mass | Diseases and traits | UKBB | -0.0258 | 7.94E-06 | 330762 |
| HRV | - | - | - | - | - | - | - | - | - | - |

Pos(h19) indicates chromosome position; A1, Effect allele; A2, Other allele; Web of PhenoScanner V2 (Date: April 8,2023):<http://www.phenoscanner.medschl.cam.ac.uk/>

Supplementary Table S5 The steiger test for the association between exposure and outcome

|  | **Outcome** | **SA** | | | |  | **TH** | | | |
| --- | --- | --- | --- | --- | --- | --- | --- | --- | --- | --- |
|  |  | **RHR** | | **HRV** | |  | **RHR** | | **HRV** | |
|  |  | **Direction** | **Steiger P** | **Direction** | **Steiger P** |  | **Direction** | **Steiger P** | **Direction** | **Steiger P** |
|  | Global | TRUE | 4.11E-185 | TRUE | 1.63E-85 |  | TRUE | 7.75E-170 | TRUE | 2.59E-70 |
| Regions with global weighted | bankssts | TRUE | 3.98E-149 | TRUE | 3.40E-69 |  | TRUE | 5.22E-185 | TRUE | 5.38E-75 |
|  | Caudal anterior cingulate | TRUE | 2.93E-169 | TRUE | 1.55E-79 |  | TRUE | 2.00E-222 | TRUE | 2.56E-79 |
|  | Caudal middle frontal | TRUE | 1.26E-192 | TRUE | 6.26E-86 |  | TRUE | 1.13E-172 | TRUE | 8.58E-75 |
|  | Cuneus | TRUE | 8.25E-180 | TRUE | 3.91E-80 |  | TRUE | 1.39E-188 | TRUE | 2.50E-88 |
|  | Entorhinal | TRUE | 1.51E-187 | TRUE | 1.18E-82 |  | TRUE | 1.31E-183 | TRUE | 6.85E-82 |
|  | Frontal pole | TRUE | 9.33E-170 | TRUE | 1.81E-74 |  | TRUE | 5.43E-168 | TRUE | 2.35E-87 |
|  | Fusiform | TRUE | 3.21E-170 | TRUE | 1.61E-71 |  | TRUE | 2.21E-183 | TRUE | 5.13E-88 |
|  | Inferior parietal | TRUE | 1.90E-194 | TRUE | 7.33E-80 |  | TRUE | 3.36E-191 | TRUE | 6.64E-80 |
|  | Inferior temporal | TRUE | 2.24E-189 | TRUE | 3.17E-80 |  | TRUE | 7.85E-173 | TRUE | 2.47E-89 |
|  | Insula | TRUE | 1.42E-178 | TRUE | 7.01E-85 |  | TRUE | 2.32E-176 | TRUE | 8.14E-85 |
|  | Isthmuscingulate | TRUE | 4.98E-170 | TRUE | 9.26E-75 |  | TRUE | 9.94E-188 | TRUE | 2.75E-77 |
|  | Lateral occipital | TRUE | 1.08E-185 | TRUE | 2.21E-80 |  | TRUE | 6.90E-179 | TRUE | 4.17E-83 |
|  | Lateral orbitofrontal | TRUE | 6.07E-175 | TRUE | 8.08E-80 |  | TRUE | 2.41E-200 | TRUE | 3.82E-86 |
|  | Lingual | TRUE | 6.03E-190 | TRUE | 3.33E-79 |  | TRUE | 1.57E-185 | TRUE | 2.24E-82 |
|  | Medial orbitofrontal | TRUE | 2.54E-187 | TRUE | 6.51E-78 |  | TRUE | 2.50E-194 | TRUE | 1.80E-82 |
|  | Middle temporal | TRUE | 6.62E-162 | TRUE | 1.10E-81 |  | TRUE | 2.84E-169 | TRUE | 3.04E-74 |
|  | Paracentral | TRUE | 3.60E-188 | TRUE | 2.00E-84 |  | TRUE | 3.04E-186 | TRUE | 4.40E-79 |
|  | Parahippocampal | TRUE | 2.69E-160 | TRUE | 6.74E-80 |  | TRUE | 1.63E-186 | TRUE | 1.25E-90 |
|  | Pars opercularis | TRUE | 6.70E-191 | TRUE | 3.51E-79 |  | TRUE | 1.24E-177 | TRUE | 1.33E-89 |
|  | Pars orbitalis | TRUE | 2.21E-179 | TRUE | 1.74E-79 |  | TRUE | 4.64E-208 | TRUE | 4.48E-85 |
|  | Pars triangularis | TRUE | 3.05E-193 | TRUE | 7.22E-69 |  | TRUE | 1.82E-182 | TRUE | 2.12E-74 |
|  | Pericalcarine | TRUE | 7.36E-175 | TRUE | 4.32E-76 |  | TRUE | 2.54E-174 | TRUE | 2.60E-80 |
|  | Postcentral | TRUE | 3.04E-165 | TRUE | 9.95E-74 |  | TRUE | 2.28E-168 | TRUE | 4.44E-85 |
|  | Posterior cingulate | TRUE | 6.87E-198 | TRUE | 1.08E-84 |  | TRUE | 8.38E-188 | TRUE | 2.76E-67 |
|  | Precentral | TRUE | 1.03E-191 | TRUE | 1.05E-81 |  | TRUE | 5.05E-171 | TRUE | 4.16E-78 |
|  | Precuneus | TRUE | 5.28E-178 | TRUE | 1.69E-68 |  | TRUE | 4.71E-183 | TRUE | 2.80E-89 |
|  | Rostral anterior cingulate | TRUE | 1.31E-190 | TRUE | 1.11E-88 |  | TRUE | 2.75E-205 | TRUE | 5.96E-86 |
|  | Rostral middle frontal | TRUE | 5.45E-177 | TRUE | 6.48E-80 |  | TRUE | 6.70E-176 | TRUE | 2.19E-87 |
|  | Superior frontal | TRUE | 2.82E-177 | TRUE | 1.27E-89 |  | TRUE | 2.08E-172 | TRUE | 1.05E-69 |
|  | Superior parietal | TRUE | 8.19E-188 | TRUE | 4.86E-81 |  | TRUE | 7.62E-194 | TRUE | 2.56E-80 |
|  | Superior temporal | TRUE | 1.97E-165 | TRUE | 1.17E-68 |  | TRUE | 4.47E-180 | TRUE | 8.15E-76 |
|  | Supramarginal | TRUE | 4.53E-174 | TRUE | 9.36E-68 |  | TRUE | 2.09E-191 | TRUE | 1.38E-79 |
|  | Temporal pole | TRUE | 3.42E-196 | TRUE | 5.39E-84 |  | TRUE | 3.82E-183 | TRUE | 2.11E-88 |
|  | Transverse temporal | TRUE | 1.11E-182 | TRUE | 2.32E-71 |  | TRUE | 6.02E-176 | TRUE | 1.38E-78 |
| Regions without global weighted | bankssts | TRUE | 1.60E-159 | TRUE | 4.20E-68 |  | TRUE | 3.27E-191 | TRUE | 8.14E-68 |
|  | Caudal anterior cingulate | TRUE | 1.64E-177 | TRUE | 1.95E-81 |  | TRUE | 2.42E-210 | TRUE | 2.32E-74 |
|  | Caudal middle frontal | TRUE | 1.40E-192 | TRUE | 9.47E-86 |  | TRUE | 1.86E-163 | TRUE | 7.55E-78 |
|  | Cuneus | TRUE | 3.24E-176 | TRUE | 5.33E-88 |  | TRUE | 2.58E-179 | TRUE | 5.76E-87 |
|  | Entorhinal | TRUE | 7.65E-184 | TRUE | 6.42E-81 |  | TRUE | 4.80E-193 | TRUE | 4.23E-82 |
|  | Frontal pole | TRUE | 4.08E-179 | TRUE | 2.51E-76 |  | TRUE | 5.96E-166 | TRUE | 5.99E-81 |
|  | Fusiform | TRUE | 2.42E-179 | TRUE | 2.78E-85 |  | TRUE | 7.21E-172 | TRUE | 1.91E-73 |
|  | Inferior parietal | TRUE | 3.54E-192 | TRUE | 3.74E-81 |  | TRUE | 2.74E-177 | TRUE | 1.28E-71 |
|  | Inferior temporal | TRUE | 4.20E-203 | TRUE | 1.17E-86 |  | TRUE | 3.02E-172 | TRUE | 3.78E-77 |
|  | Insula | TRUE | 1.22E-168 | TRUE | 5.98E-79 |  | TRUE | 4.75E-181 | TRUE | 9.40E-82 |
|  | Isthmuscingulate | TRUE | 2.93E-171 | TRUE | 4.00E-79 |  | TRUE | 3.27E-178 | TRUE | 2.64E-71 |
|  | Lateral occipital | TRUE | 5.01E-172 | TRUE | 2.38E-82 |  | TRUE | 1.39E-178 | TRUE | 1.93E-74 |
|  | Lateral orbitofrontal | TRUE | 6.94E-180 | TRUE | 4.58E-81 |  | TRUE | 1.52E-178 | TRUE | 8.36E-76 |
|  | Lingual | TRUE | 3.16E-196 | TRUE | 2.61E-81 |  | TRUE | 1.09E-175 | TRUE | 1.77E-77 |
|  | Medial orbitofrontal | TRUE | 1.28E-174 | TRUE | 9.76E-89 |  | TRUE | 9.42E-189 | TRUE | 1.28E-79 |
|  | Middle temporal | TRUE | 2.49E-178 | TRUE | 3.54E-81 |  | TRUE | 4.92E-177 | TRUE | 4.28E-73 |
|  | Paracentral | TRUE | 2.51E-181 | TRUE | 2.06E-88 |  | TRUE | 9.18E-178 | TRUE | 1.42E-70 |
|  | Parahippocampal | TRUE | 4.96E-176 | TRUE | 4.01E-78 |  | TRUE | 8.10E-185 | TRUE | 2.96E-87 |
|  | Pars opercularis | TRUE | 3.04E-189 | TRUE | 3.30E-80 |  | TRUE | 7.74E-174 | TRUE | 3.00E-76 |
|  | Pars orbitalis | TRUE | 2.79E-180 | TRUE | 3.32E-82 |  | TRUE | 3.86E-191 | TRUE | 9.97E-78 |
|  | Pars triangularis | TRUE | 7.07E-187 | TRUE | 1.23E-72 |  | TRUE | 5.78E-175 | TRUE | 4.59E-70 |
|  | Pericalcarine | TRUE | 1.66E-180 | TRUE | 9.75E-88 |  | TRUE | 4.09E-175 | TRUE | 6.64E-78 |
|  | Postcentral | TRUE | 4.83E-161 | TRUE | 1.69E-73 |  | TRUE | 3.34E-169 | TRUE | 1.28E-88 |
|  | Posterior cingulate | TRUE | 1.03E-190 | TRUE | 2.54E-88 |  | TRUE | 2.90E-170 | TRUE | 3.68E-65 |
|  | Precentral | TRUE | 1.59E-186 | TRUE | 1.77E-82 |  | TRUE | 1.99E-181 | TRUE | 5.91E-82 |
|  | Precuneus | TRUE | 1.27E-188 | TRUE | 1.72E-90 |  | TRUE | 3.90E-159 | TRUE | 3.26E-73 |
|  | Rostral anterior cingulate | TRUE | 7.60E-194 | TRUE | 1.66E-89 |  | TRUE | 2.73E-201 | TRUE | 1.82E-88 |
|  | Rostral middle frontal | TRUE | 6.49E-192 | TRUE | 1.47E-79 |  | TRUE | 1.99E-171 | TRUE | 4.53E-75 |
|  | Superior frontal | TRUE | 2.43E-183 | TRUE | 1.36E-83 |  | TRUE | 3.25E-162 | TRUE | 1.22E-72 |
|  | Superior parietal | TRUE | 7.00E-181 | TRUE | 6.17E-86 |  | TRUE | 5.79E-166 | TRUE | 3.04E-70 |
|  | Superior temporal | TRUE | 6.49E-152 | TRUE | 2.56E-67 |  | TRUE | 7.03E-161 | TRUE | 8.55E-73 |
|  | Supramarginal | TRUE | 8.57E-159 | TRUE | 3.37E-68 |  | TRUE | 4.53E-171 | TRUE | 1.01E-69 |
|  | Temporal pole | TRUE | 2.01E-181 | TRUE | 3.11E-79 |  | TRUE | 5.09E-194 | TRUE | 1.02E-88 |
|  | Transverse temporal | TRUE | 1.58E-186 | TRUE | 2.79E-73 |  | TRUE | 3.32E-173 | TRUE | 3.58E-78 |

RHR, resting heart rate; HRV, heart rate variability; SA, cortical surface area; TH, cortical thickness

Supplementary Table S6 Significant and nominal significant all MR estimates from RHR and HRV on genetically predicted cortical structure

|  | **Exposure** |  | **MR analysis methods** | **beta** | **se** | **P-value** | **Q** | **heterogeneity** | **egger_intercept** | **pleiotropy** | **Distortion Test** |
| --- | --- | --- | --- | --- | --- | --- | --- | --- | --- | --- | --- |
|  |  | Global SA | Inverse variance weighted | -61.64806 | 34.78528 | 0.0763538 | 77.53831 | 0.4282964 | 59.42308 | 0.04645522 |  |
|  |  |  | MR Egger | -185.20146 | 70.1134 | 0.0099473 | 81.63087 | 0.525459 |  |  |  |
|  |  |  | Weighted median | -152.8897 | 52.44536 | 0.0035544 |  |  |  |  |  |
|  |  |  | MR-PRESSO | NA | NA | NA | NA | NA | NA | NA | NA |
| With global weighted | RHR | SA of entorhinal | Inverse variance weighted | 0.3254282 | 0.1509107 | 0.03105017 | 77.91435 | 0.5451786 | -0.211 | 0.1051405 |  |
|  |  |  | MR Egger | 0.7642532 | 0.3073072 | 0.01499248 | 75.227 | 0.5993931 |  |  |  |
|  |  |  | Weighted median | 0.4476387 | 0.2281612 | 0.04976939 |  |  |  |  |  |
|  |  |  | MR-PRESSO | NA | NA | NA | NA | NA | NA | NA | NA |
|  | RHR | SA of inferiortemporal | Inverse variance weighted | 1.375977 | 0.6972367 | 0.048441908 | 84.49459 | 0.3440709 | -1.33 | 0.02420579 |  |
|  |  |  | MR Egger | 4.143097 | 1.3825251 | 0.003645199 | 79.2 | 0.4725035 |  |  |  |
|  |  |  | Weighted median | 3.218585 | 1.0300367 | 0.001779695 |  |  |  |  |  |
|  |  |  | MR-PRESSO | NA | NA | NA | NA | NA | NA | NA | NA |
|  | RHR | SA of fusiform | Inverse variance weighted | 1.74885 | 0.714909 | 0.01443489 | 114.7557 | 0.00659353 | 0.142821 | 0.8165077 |  |
|  |  |  | MR Egger | 1.452118 | 1.4634418 | 0.32409906 | 114.67 | 0.0054094 |  |  |  |
|  |  |  | Weighted median | 1.589024 | 0.9262237 | 0.08623635 |  |  |  |  |  |
|  |  |  | MR-PRESSO | NA | NA | NA | NA | NA | NA | NA | NA |
|  | RHR | SA of lingual | Inverse variance weighted | 1.4938146 | 0.7376336 | 0.04285253 | 83.02826 | 0.3862825 | -0.491 | 0.4385749 |  |
|  |  |  | MR Egger | 2.5153145 | 1.5060924 | 0.0988588 | 82.39608 | 0.3747563 |  |  |  |
|  |  |  | Weighted median | 0.0028788 | 1.1095325 | 0.99792984 |  |  |  |  |  |
|  |  |  | MR-PRESSO | NA | NA | NA | NA | NA | NA | NA | NA |
|  | RHR | SA of paracentral | Inverse variance weighted | 0.7873104 | 0.3814181 | 0.03900195 | 92.7684 | 0.1556971 | 0.177 | 0.589174 |  |
|  |  |  | MR Egger | 0.418897 | 0.7799901 | 0.59273958 | 92.4244 | 0.1433884 |  |  |  |
|  |  |  | Weighted median | 0.7802589 | 0.5298899 | 0.14088795 |  |  |  |  |  |
|  |  |  | MR-PRESSO | NA | NA | NA | NA | NA | NA | NA | NA |
|  | RHR | SA of parstriangularis | Inverse variance weighted | -0.3841528 | 0.3895503 | 0.32406231 | 74.62255 | 0.6487714 | 0.752 | 0.02638958 |  |
|  |  |  | MR Egger | -1.9483952 | 0.7934725 | 0.01626344 | 69.50199 | 0.7687093 |  |  |  |
|  |  |  | Weighted median | -1.4311054 | 0.5951367 | 0.01618721 |  |  |  |  |  |
|  |  |  | MR-PRESSO | NA | NA | NA | NA | NA | NA | NA | NA |
|  | RHR | SA of precentral | Inverse variance weighted | 0.7318692 | 0.7978348 | 0.35897539 | 77.85647 | 0.5470232 | -1.38 | 0.04578025 |  |
|  |  |  | MR Egger | 3.601461 | 1.6235368 | 0.02940689 | 73.73782 | 0.6461481 |  |  |  |
|  |  |  | Weighted median | 2.0777774 | 1.1720082 | 0.07625601 |  |  |  |  |  |
|  |  |  | MR-PRESSO | NA | NA | NA | NA | NA | NA | NA | NA |
|  | RHR | SA of rostralmiddlefrontal | Inverse variance weighted | -3.257091 | 1.079255 | 0.002545263 | 101.6829 | 0.05138097 | 3.16E-01 | 0.7337635 |  |
|  |  |  | MR Egger | -3.913836 | 2.209057 | 0.0802967 | 101.5332 | 0.04476849 |  |  |  |
|  |  |  | Weighted median | -3.224576 | 1.43023 | 0.024159366 |  |  |  |  |  |
|  |  |  | MR-PRESSO | NA | NA | NA | NA | NA | NA | NA | NA |
|  | RHR | SA of superiortemporal | Inverse variance weighted | -1.674542 | 0.6352732 | 0.008390459 | 101.1641 | 0.05518174 | 3.58E-01 | 0.5119017 |  |
|  |  |  | MR Egger | -2.419108 | 1.2975113 | 0.065976803 | 100.6112 | 0.05096714 |  |  |  |
|  |  |  | Weighted median | -1.314015 | 0.8971872 | 0.143031644 |  |  |  |  |  |
|  |  |  | MR-PRESSO | NA | NA | NA | NA | NA | NA | NA | NA |
|  | RHR | SA of temporalpole | Inverse variance weighted | -0.2960112 | 0.1139473 | 0.009382577 | 66.52708 | 0.8594986 | 5.77E-02 | 0.553978 |  |
|  |  |  | MR Egger | -0.4160925 | 0.2319563 | 0.076664965 | 66.17383 | 0.8479541 |  |  |  |
|  |  |  | Weighted median | -0.4687862 | 0.1571004 | 0.002845149 |  |  |  |  |  |
|  |  |  | MR-PRESSO | NA | NA | NA | NA | NA | NA | NA | NA |
|  | RHR | TH of bankssts | Inverse variance weighted | 0.001152 | 0.0002968 | 0.00010405 | 72.25104 | 0.7191824 | -4.95E-04 | 0.05358335 |  |
|  |  |  | MR Egger | 0.0021944 | 0.0006092 | 0.000549791 | 68.41145 | 0.796611 |  |  |  |
|  |  |  | Weighted median | 0.0015672 | 0.0004328 | 0.00029291 |  |  |  |  |  |
|  |  |  | MR-PRESSO | NA | NA | NA | NA | NA | NA | NA | NA |
|  | RHR | TH of caudalanteriorcingulate | Inverse variance weighted | 0.0009847 | 0.000486 | 0.04272629 | 50.27608 | 0.9962332 | -1.77E-04 | 0.670018 |  |
|  |  |  | MR Egger | 0.0013525 | 0.0009877 | 0.17475518 | 50.09313 | 0.9954254 |  |  |  |
|  |  |  | Weighted median | 0.0015637 | 0.0006854 | 0.02252172 |  |  |  |  |  |
|  |  |  | MR-PRESSO | NA | NA | NA | NA | NA | NA | NA | NA |
|  | RHR | TH of fusiform | Inverse variance weighted | 0.0002254 | 0.000282 | 0.42424895 | 103.545 | 0.03950697 | -5.49E-04 | 0.02254654 |  |
|  |  |  | MR Egger | 0.0013523 | 0.0005568 | 0.01741708 | 96.90476 | 0.0835994 |  |  |  |
|  |  |  | Weighted median | 0.0006587 | 0.0003833 | 0.08568994 |  |  |  |  |  |
|  |  |  | MR-PRESSO | NA | NA | NA | NA | NA | NA | NA | NA |
|  | RHR | TH of inferiorparietal | Inverse variance weighted | 0.0005013 | 0.0001878 | 0.007589893 | 84.93947 | 0.3317046 | -1.60E-04 | 0.3193721 |  |
|  |  |  | MR Egger | 0.0008318 | 0.0003795 | 0.031330456 | 83.8734 | 0.3325757 |  |  |  |
|  |  |  | Weighted median | 0.0010809 | 0.0002871 | 0.000167083 |  |  |  |  |  |
|  |  |  | MR-PRESSO | NA | NA | NA | NA | NA | NA | NA | NA |
|  | RHR | TH of lateraloccipital | Inverse variance weighted | 0.0005521 | 0.0002423 | 0.022699566 | 100.0907 | 0.06379413 | -5.29E-04 | 0.00940834 |  |
|  |  |  | MR Egger | 0.0016469 | 0.000473 | 0.000814191 | 91.85181 | 0.15289192 |  |  |  |
|  |  |  | Weighted median | 0.0007252 | 0.0003452 | 0.035655504 |  |  |  |  |  |
|  |  |  | MR-PRESSO | NA | NA | NA | NA | NA | NA | NA | NA |
|  | RHR | TH of medialorbitofrontal | Inverse variance weighted | -0.0007198 | 0.0003223 | 0.02551731 | 75.57259 | 0.619325 | -7.00E-05 | 0.8001757 |  |
|  |  |  | MR Egger | -0.0005732 | 0.000661 | 0.38845986 | 75.50809 | 0.5904633 |  |  |  |
|  |  |  | Weighted median | -0.0006595 | 0.0004695 | 0.16009823 |  |  |  |  |  |
|  |  |  | MR-PRESSO | NA | NA | NA | NA | NA | NA | NA | NA |
|  | RHR | TH of paracentral | Inverse variance weighted | -0.0005679 | 0.0002941 | 0.05351712 | 96.51321 | 0.1007981 | 8.85E-04 | 0.000235655 |  |
|  |  |  | MR Egger | -0.0024175 | 0.0005515 | 3.55716E-05 | 81.24192 | 0.4091848 |  |  |  |
|  |  |  | Weighted median | -0.0013644 | 0.0004077 | 0.000818014 |  |  |  |  |  |
|  |  |  | MR-PRESSO | NA | NA | NA | NA | NA | NA | NA | NA |
|  | RHR | TH of parsopercularis | Inverse variance weighted | -0.0005655 | 0.0002611 | 0.03034969 | 110.4201 | 0.01372562 | 3.11E-04 | 0.1621771 |  |
|  |  |  | MR Egger | -0.0012131 | 0.0005273 | 0.02404849 | 107.7059 | 0.01761423 |  |  |  |
|  |  |  | Weighted median | -0.0006278 | 0.0003344 | 0.06042306 |  |  |  |  |  |
|  |  |  | MR-Presso(2 outliers) | -0.0006955 | 0.0002178 | 0.002030184 |  |  |  |  | 0.535 |
|  | RHR | TH of parstriangularis | Inverse variance weighted | 0.0005207 | 0.0002879 | 0.070458437 | 110.5618 | 0.01341114 | -6.20E-04 | 0.01009919 |  |
|  |  |  | MR Egger | 0.0018212 | 0.0005662 | 0.001880471 | 101.6244 | 0.04419184 |  |  |  |
|  |  |  | Weighted median | 0.0009559 | 0.0003724 | 0.010263814 |  |  |  |  |  |
|  |  |  | MR-PRESSO | NA | NA | NA | NA | NA | NA | NA | NA |
|  | RHR | TH of postcentral | Inverse variance weighted | -0.0007374 | 0.0002429 | 0.002402063 | 102.6883 | 0.04464199 | 1.27E-04 | 0.5437981 |  |
|  |  |  | MR Egger | -0.0010001 | 0.0004951 | 0.046760821 | 102.2073 | 0.04065264 |  |  |  |
|  |  |  | Weighted median | -0.0010574 | 0.0003236 | 0.001084782 |  |  |  |  |  |
|  |  |  | MR-PRESSO | NA | NA | NA | NA | NA | NA | NA | NA |
|  | RHR | TH of posteriorcingulate | Inverse variance weighted | 0.0007753 | 0.0003023 | 0.01033547 | 88.18426 | 0.2487305 | -1.91E-04 | 0.4600761 |  |
|  |  |  | MR Egger | 0.0011757 | 0.0006187 | 0.0610394 | 87.57337 | 0.2384037 |  |  |  |
|  |  |  | Weighted median | 0.0002594 | 0.0004402 | 0.5557186 |  |  |  |  |  |
|  |  |  | MR-PRESSO | NA | NA | NA | NA | NA | NA | NA | NA |
|  | RHR | TH of precentral | Inverse variance weighted | -0.000502 | 0.0002851 | 0.078323217 | 120.0168 | 0.00700077 | 5.16E-04 | 0.03236738 |  |
|  |  |  | MR Egger | -0.0015888 | 0.0005715 | 0.006789946 | 113.2168 | 0.00254018 |  |  |  |
|  |  |  | Weighted median | -0.0002454 | 0.0003657 | 0.502284932 |  |  |  |  |  |
|  |  |  | MR-PRESSO(1 outlier) | -0.0005592 | 0.0002743 | 0.044826 |  |  |  |  | 0.832 |
|  | RHR | TH of superiorfrontal | Inverse variance weighted | 0.0005947 | 0.0002311 | 0.010061869 | 97.41818 | 0.09012714 | -5.41E-05 | 0.7868207 |  |
|  |  |  | MR Egger | 0.000708 | 0.0004777 | 0.142347848 | 97.32746 | 0.07918448 |  |  |  |
|  |  |  | Weighted median | 0.0010125 | 0.0003456 | 0.003392434 |  |  |  |  |  |
|  |  |  | MR-PRESSO | NA | NA | NA | NA | NA | NA | NA | NA |
|  | RHR | TH of temporalpole | Inverse variance weighted | 0.0017147 | 0.0006995 | 0.014234538 | 90.67748 | 0.1945018 | -1.41E-03 | 0.01643412 |  |
|  |  |  | MR Egger | 0.0046623 | 0.0013806 | 0.001139778 | 84.26704 | 0.3217401 |  |  |  |
|  |  |  | Weighted median | 0.0018275 | 0.0010693 | 0.087434825 |  |  |  |  |  |
|  |  |  | MR-PRESSO | NA | NA | NA | NA | NA | NA | NA | NA |
|  | HRV | SA of temporalpole | Inverse variance weighted | 3.853436 | 1.73259 | 0.0261424 | 7.89914 | 0.9277626 | 2.85E-02 | 0.8607217 |  |
|  |  |  | MR Egger | 3.51253 | 2.576932 | 0.1943832 | 7.867201 | 0.8961291 |  |  |  |
|  |  |  | Weighted median | 3.064861 | 2.301011 | 0.1828724 |  |  |  |  |  |
|  |  |  | MR-PRESSO | NA | NA | NA | NA | NA | NA | NA | NA |
|  | HRV | TH of caudalanteriorcingulate | Inverse variance weighted | -0.0180407 | 0.0073759 | 0.01444981 | 13.41474 | 0.5702965 | 7.25E-04 | 0.3050201 |  |
|  |  |  | MR Egger | -0.0266197 | 0.0109237 | 0.02876085 | 12.28111 | 0.583739 |  |  |  |
|  |  |  | Weighted median | -0.0191283 | 0.0098932 | 0.05317745 |  |  |  |  |  |
|  |  |  | MR-PRESSO | NA | NA | NA | NA | NA | NA | NA | NA |
|  | HRV | TH of superiortemporal | Inverse variance weighted | 0.0080212 | 0.0038762 | 0.03851514 | 15.03147 | 0.4491528 | 3.91E-04 | 0.2967141 |  |
|  |  |  | MR Egger | 0.0034108 | 0.0057521 | 0.56266159 | 13.85658 | 0.4604496 |  |  |  |
|  |  |  | Weighted median | 0.0034685 | 0.0051318 | 0.49911704 |  |  |  |  |  |
|  |  |  | MR-PRESSO | NA | NA | NA | NA | NA | NA | NA | NA |
|  | HRV | TH of supramarginal | Inverse variance weighted | -0.0056223 | 0.0028183 | 0.04604557 | 13.92199 | 0.5314537 | 1.17E-04 | 0.6602409 |  |
|  |  |  | MR Egger | -0.0070277 | 0.0042114 | 0.11736968 | 13.72031 | 0.4707495 |  |  |  |
|  |  |  | Weighted median | -0.0031479 | 0.0038829 | 0.41753629 |  |  |  |  |  |
|  |  |  | MR-PRESSO | NA | NA | NA | NA | NA | NA | NA | NA |
| Without global weighted | RHR | SA of caudalanteriorcingulate | Inverse variance weighted | -0.7045737 | 0.3096441 | 0.0228801 | 90.02237 | 0.2079133 | 0.254647 | 0.3379363 |  |
|  |  |  | MR Egger | -1.2343187 | 0.6307779 | 0.0539017 | 88.9755 | 0.2075358 |  |  |  |
|  |  |  | Weighted median | -1.1121902 | 0.4093163 | 0.0065839 |  |  |  |  |  |
|  |  |  | MR-PRESSO | NA | NA | NA | NA | NA | NA | NA | NA |
|  | RHR | SA of insula | Inverse variance weighted | -1.120497 | 0.6964781 | 0.1076592 | 114.4828 | 0.001943479 | 1.008547 | 0.03150193 |  |
|  |  |  | MR Egger | -3.764285 | 1.3859882 | 0.008115 | 121.4307 | 0.00559993 |  |  |  |
|  |  |  | Weighted median | -3.226911 | 0.8744987 | 0.0002242 |  |  |  |  |  |
|  |  |  | MR-presso(2 outliers) | -1.633391 | 0.6014438 | 0.0081391 |  |  |  |  | 0.528 |
|  | RHR | SA of isthmuscingulate | Inverse variance weighted | -0.6733568 | 0.4264277 | 0.1143213 | 87.32164 | 0.2202321 | 0.486556 | 0.1350559 |  |
|  |  |  | MR Egger | -1.9840534 | 0.8575763 | 0.0232929 | 84.80925 | 0.2538238 |  |  |  |
|  |  |  | Weighted median | -1.4875969 | 0.5592781 | 0.0078175 |  |  |  |  |  |
|  |  |  | MR-PRESSO(2 outliers) | -0.9527428 | 0.3836323 | 0.0151557 |  |  |  |  | 0.595 |
|  | RHR | SA of middletemporal | Inverse variance weighted | -2.325913 | 0.9348866 | 0.0128497 | 83.1649 | 0.3822616 | 1.46866 | 0.06505817 |  |
|  |  |  | MR Egger | -5.381815 | 1.8749403 | 0.0052589 | 79.63634 | 0.458774 |  |  |  |
|  |  |  | Weighted median | -2.977106 | 1.3866737 | 0.031798 |  |  |  |  |  |
|  |  |  | MR-PRESSO | NA | NA | NA | NA | NA | NA | NA | NA |
|  | RHR | SA of parsopercularis | Inverse variance weighted | -0.1585239 | 0.5364534 | 0.7676092 | 87.61511 | 0.2623064 | 0.9673723 | 0.03354316 |  |
|  |  |  | MR Egger | -2.172607 | 1.0686222 | 0.045402 | 82.71528 | 0.365451 |  |  |  |
|  |  |  | Weighted median | -0.8897899 | 0.8262875 | 0.2815461 |  |  |  |  |  |
|  |  |  | MR-PRESSO | NA | NA | NA | NA | NA | NA | NA | NA |
|  | RHR | SA of parsorbitalis | Inverse variance weighted | -0.4051494 | 0.2184649 | 0.0636636 | 103.1039 | 0.04208425 | 0.365421 | 0.04915661 |  |
|  |  |  | MR Egger | -1.1656392 | 0.4368983 | 0.0092556 | 98.1444 | 0.07118738 |  |  |  |
|  |  |  | Weighted median | -0.3876477 | 0.294719 | 0.1884048 |  |  |  |  |  |
|  |  |  | MR-PRESSO | NA | NA | NA | NA | NA | NA | NA | NA |
|  | RHR | SA of parstriangularis | Inverse variance weighted | -1.05116 | 0.4810428 | 0.0288764 | 88.97444 | 0.2306118 | 1.266249 | 0.001665498 |  |
|  |  |  | MR Egger | -3.685526 | 0.9288034 | 0.0001585 | 78.37295 | 0.4987689 |  |  |  |
|  |  |  | Weighted median | -1.220878 | 0.7176188 | 0.0888885 |  |  |  |  |  |
|  |  |  | MR-PRESSO | NA | NA | NA | NA | NA | NA | NA | NA |
|  | RHR | SA of posteriorcingulate | Inverse variance weighted | -0.7616737 | 0.3713168 | 0.04024 | 78.10389 | 0.5391375 | 0.740255 | 0.02190849 |  |
|  |  |  | MR Egger | -2.3028824 | 0.7565411 | 0.0031705 | 72.63682 | 0.6797726 |  |  |  |
|  |  |  | Weighted median | -1.6069529 | 0.5380858 | 0.0028226 |  |  |  |  |  |
|  |  |  | MR-PRESSO | NA | NA | NA | NA | NA | NA | NA | NA |
|  | RHR | SA of rostralmiddlefrontal | Inverse variance weighted | -5.746372 | 1.662474 | 0.0005472 | 61.94783 | 0.9328691 | 3.00913 | 0.03687572 |  |
|  |  |  | MR Egger | -12.010849 | 3.386736 | 0.0006601 | 57.44026 | 0.9676735 |  |  |  |
|  |  |  | Weighted median | -9.69816 | 2.553902 | 0.0001462 |  |  |  |  |  |
|  |  |  | MR-PRESSO | NA | NA | NA | NA | NA | NA | NA | NA |
|  | RHR | SA of superiortemporal | Inverse variance weighted | -2.896457 | 1.129911 | 0.0103642 | 114.0245 | 0.00748703 | 1.624154 | 0.09195822 |  |
|  |  |  | MR Egger | -6.275661 | 2.273968 | 0.0071865 | 109.9735 | 0.0121693 |  |  |  |
|  |  |  | Weighted median | -5.088054 | 1.412567 | 0.0003158 |  |  |  |  |  |
|  |  |  | MR-PRESSO | NA | NA | NA | NA | NA | NA | NA | NA |
|  | RHR | SA of supramarginal | Inverse variance weighted | -3.364226 | 1.462004 | 0.0213857 | 127.5909 | 0.00057387 | 1.950141 | 0.1184167 |  |
|  |  |  | MR Egger | -7.421346 | 2.950143 | 0.0139115 | 123.6891 | 0.00098125 |  |  |  |
|  |  |  | Weighted median | -6.360267 | 1.848135 | 0.0005786 |  |  |  |  |  |
|  |  |  | MR-PRESSO(3 outliers) | -3.647117 | 1.313457 | 0.0068916 |  |  |  |  | 0.818 |
|  | RHR | SA of temporalpole | Inverse variance weighted | -0.4234327 | 0.1342883 | 1.62E-03 | 91.83224 | 0.1723217 | 0.189331 | 0.09845108 |  |
|  |  |  | MR Egger | -0.8175603 | 0.2705409 | 3.38E-03 | 88.69308 | 0.2135308 |  |  |  |
|  |  |  | Weighted median | -0.7978744 | 0.1924312 | 3.38E-05 |  |  |  |  |  |
|  |  |  | MR-PRESSO | NA | NA | NA | NA | NA | NA | NA | NA |
|  | RHR | SA of transversetemporal | Inverse variance weighted | -0.3909373 | 0.1630406 | 0.0164943 | 96.42771 | 0.10185551 | -0.105355 | 0.4519897 |  |
|  |  |  | MR Egger | -0.171766 | 0.332877 | 0.6072925 | 95.73538 | 0.09684935 |  |  |  |
|  |  |  | Weighted median | -0.1514316 | 0.2320688 | 0.5140599 |  |  |  |  |  |
|  |  |  | MR-PRESSO | NA | NA | NA | NA | NA | NA | NA | NA |
|  | RHR | TH of bankssts | Inverse variance weighted | 0.0010351 | 0.0003866 | 0.0074141 | 65.93831 | 0.8709468 | -0.000659 | 0.04905192 |  |
|  |  |  | MR Egger | 0.0024107 | 0.0007893 | 0.0030749 | 61.94247 | 0.9214829 |  |  |  |
|  |  |  | Weighted median | 0.0014248 | 0.0005603 | 0.0109933 |  |  |  |  |  |
|  |  |  | MR-PRESSO | NA | NA | NA | NA | NA | NA | NA | NA |
|  | RHR | TH of lateraloccipital | Inverse variance weighted | 0.0005081 | 0.0003617 | 0.1601571 | 106.8683 | 0.02409863 | -0.000683 | 0.02469323 |  |
|  |  |  | MR Egger | 0.0019325 | 0.000715 | 0.0084122 | 100.2162 | 0.05383678 |  |  |  |
|  |  |  | Weighted median | 0.0007805 | 0.0004778 | 0.1023562 |  |  |  |  |  |
|  |  |  | MR-PRESSO(1 outlier) | 0.0007395 | 0.0003219 | 0.0242545 |  |  |  |  | 0.790 |
|  | RHR | TH of paracentral | Inverse variance weighted | -0.0006767 | 0.0004228 | 0.1094793 | 110.17 | 0.08971336 | 0.000746 | 0.03700131 |  |
|  |  |  | MR Egger | -0.0022438 | 0.0008466 | 0.0097144 | 104.231 | 0.1764778 |  |  |  |
|  |  |  | Weighted median | -0.0018915 | 0.0005687 | 0.0008806 |  |  |  |  |  |
|  |  |  | MR-PRESSO | NA | NA | NA | NA | NA | NA | NA | NA |
|  | RHR | TH of parstriangularis | Inverse variance weighted | 0.0005043 | 0.0004129 | 0.2219373 | 119.9476 | 0.00257337 | -0.000779 | 0.02573342 |  |
|  |  |  | MR Egger | 0.0021177 | 0.000816 | 0.0112625 | 112.5837 | 0.00781548 |  |  |  |
|  |  |  | Weighted median | 0.0002137 | 0.0005375 | 0.6909153 |  |  |  |  |  |
|  |  |  | MR-PRESSO | NA | NA | NA | NA | NA | NA | NA | NA |
|  | RHR | TH of temporalpole | Inverse variance weighted | 0.001462 | 0.0007273 | 0.044405 | 71.99081 | 0.7265628 | -0.001632 | 0.01021136 |  |
|  |  |  | MR Egger | 0.0048642 | 0.0014833 | 0.0015488 | 65.06461 | 0.8702542 |  |  |  |
|  |  |  | Weighted median | 0.0027385 | 0.0010622 | 0.0099354 |  |  |  |  |  |
|  |  |  | MR-PRESSO | NA | NA | NA | NA | NA | NA | NA | NA |
|  | HRV | SA of temporalpole | Inverse variance weighted | 5.946455 | 1.902005 | 0.0017695 | 8.740368 | 0.8906967 | -0.035473 | 0.8422267 |  |
|  |  |  | MR Egger | 6.371003 | 2.828616 | 0.0408732 | 8.699249 | 0.8498207 |  |  |  |
|  |  |  | Weighted median | 5.544058 | 2.531887 | 0.0285464 |  |  |  |  |  |
|  |  |  | MR-PRESSO | NA | NA | NA | NA | NA | NA | NA | NA |
|  | HRV | SA of lingual | Inverse variance weighted | 28.26765 | 13.70493 | 0.0391517 | 12.69017 | 0.6262155 | 0.6973 | 0.5887154 |  |
|  |  |  | MR Egger | 19.91994 | 20.38038 | 0.3449556 | 12.38391 | 0.5755014 |  |  |  |
|  |  |  | Weighted median | 22.57321 | 17.78747 | 0.2044229 |  |  |  |  |  |
|  |  |  | MR-PRESSO | NA | NA | NA | NA | NA | NA | NA | NA |
|  | HRV | TH of caudalanteriorcingulate | Inverse variance weighted | -0.0242993 | 0.0079591 | 0.0022654 | 15.7902 | 0.3961306 | 6.00E-05 | 0.9375677 |  |
|  |  |  | MR Egger | -0.0250251 | 0.0122748 | 0.0608238 | 15.78303 | 0.3268033 |  |  |  |
|  |  |  | Weighted median | -0.0190827 | 0.0100916 | 0.0586307 |  |  |  |  |  |
|  |  |  | MR-PRESSO | NA | NA | NA | NA | NA | NA | NA | NA |
|  | HRV | TH of posteriorcingulate | Inverse variance weighted | -0.0169646 | 0.0077039 | 0.0276585 | 33.20911 | 0.00439098 | 2.70E-06 | 0.9971137 |  |
|  |  |  | MR Egger | -0.016997 | 0.0118693 | 0.1740859 | 33.20908 | 0.00268754 |  |  |  |
|  |  |  | Weighted median | -0.0150016 | 0.0069396 | 0.0306388 |  |  |  |  |  |
|  |  |  | MR-PRESSO(1 outlier) | -0.0193092 | 0.0060619 | 0.0066102 |  |  |  |  | 0.727 |
|  | HRV | TH of superiorfrontal | Inverse variance weighted | -0.0127759 | 0.0064191 | 0.0465591 | 21.33759 | 0.1263856 | -0.000429 | 0.4795157 |  |
|  |  |  | MR Egger | -0.0075023 | 0.0097591 | 0.4548136 | 20.56242 | 0.1133831 |  |  |  |
|  |  |  | Weighted median | -0.0031377 | 0.0076578 | 0.6819994 |  |  |  |  |  |
|  |  |  | MR-PRESSO | NA | NA | NA | NA | NA | NA | NA | NA |

Heterogeneity and pleiotropy P value < 0.05 is significant. Distortion Test: P > 0.05 no difference between pre- and post-corrective outcomes HRV, heart rate variability; RHR, resting heart rate; SA, cortical surface area; TH, cortical thickness; NA, not available

Supplementary Table S7 STROBE-MR checklist of recommended items to address in reports of Mendelian randomization studies^1^ ^2^

| **Item No.** | **Section** | **Checklist item** | **Page No.** | **Relevant text from manuscript** |
| --- | --- | --- | --- | --- |
| 1 | **TITLE and ABSTRACT** | Indicate Mendelian randomization (MR) as the study’s design in the title and/or the abstract if that is a main purpose of the study | 1,2 | Complete |
|  | **INTRODUCTION** |  |  |  |
| 2 | **Background** | Explain the scientific background and rationale for the reported study. What is the exposure? Is a potential causal relationship between exposure and outcome plausible? Justify why MR is a helpful method to address the study question | 3 | Complete - causal relationship between hear rate variation and brain structure in 1-2 paragraph of introduction. |
| 3 | **Objectives** | State specific objectives clearly, including pre-specified causal hypotheses (if any). State that MR is a method that, under specific assumptions, intends to estimate causal effects |  | Complete - In the paragraph 3 of the introduction. |
|  | **METHODS** |  |  |  |
| 4 | **Study design and data sources** | Present key elements of the study design early in the article. Consider including a table listing sources of data for all phases of the study. For each data source contributing to the analysis, describe the following: |  |  |
|  | a) | Setting: Describe the study design and the underlying population, if possible. Describe the setting, locations, and relevant dates, including periods of recruitment, exposure, follow-up, and data collection, when available. | 5 | Complete - In “Data sources for RHR and HRV” and “Data source for cerebral cortex SA and TH” section. |
|  | b) | Participants: Give the eligibility criteria, and the sources and methods of selection of participants. Report the sample size, and whether any power or sample size calculations were carried out prior to the main analysis | 5 | Complete - In “Data sources for RHR and HRV” and “Data source for cerebral cortex SA and TH” section. |
|  | c) | Describe measurement, quality control and selection of genetic variants | 5 | Complete - In “Data sources for RHR and HRV” and “Data source for cerebral cortex SA and TH” section. |
|  | d) | For each exposure, outcome, and other relevant variables, describe methods of assessment and diagnostic criteria for diseases | NA |  |
|  | e) | Provide details of ethics committee approval and participant informed consent, if relevant | NA |  |
| 5 | **Assumptions** | Explicitly state the three core IV assumptions for the main analysis (relevance, independence and exclusion restriction) as well assumptions for any additional or sensitivity analysis | 4 | Complete – “Study design” section. |
| 6 | **Statistical methods: main analysis** | Describe statistical methods and statistics used |  |  |
|  | a) | Describe how quantitative variables were handled in the analyses (i.e., scale, units, model) | NA |  |
|  | b) | Describe how genetic variants were handled in the analyses and, if applicable, how their weights were selected | 6 | Complete – In “Statistical analysis” section. |
|  | c) | Describe the MR estimator (e.g. two-stage least squares, Wald ratio) and related statistics. Detail the included covariates and, in case of two-sample MR, whether the same covariate set was used for adjustment in the two samples | 6 | Complete – In “Statistical analysis” section. |
|  | d) | Explain how missing data were addressed | NA |  |
|  | e) | If applicable, indicate how multiple testing was addressed | 6 | Complete – In “Statistical analysis” section. |
| 7 | **Assessment of assumptions** | Describe any methods or prior knowledge used to assess the assumptions or justify their validity | 6 | Complete – In “Statistical analysis” section. |
| 8 | **Sensitivity analyses and additional analyses** | Describe any sensitivity analyses or additional analyses performed (e.g. comparison of effect estimates from different approaches, independent replication, bias analytic techniques, validation of instruments, simulations) | 6 | Complete – In “Statistical analysis” section. |
| 9 | **Software and pre-registration** |  |  |  |
|  | a) | Name statistical software and package(s), including version and settings used | 6-7 | Complete –The package and settings used are described in “Statistical analysis” section. |
|  | b) | State whether the study protocol and details were pre-registered (as well as when and where) | NA |  |
|  | **RESULTS** |  |  |  |
| 10 | **Descriptive data** |  |  |  |
|  | a) | Report the numbers of individuals at each stage of included studies and reasons for exclusion. Consider use of a flow diagram | 5 | Complete - In “Data sources for RHR and HRV” and “Data source for cerebral cortex SA and TH” section. |
|  | b) | Report summary statistics for phenotypic exposure(s), outcome(s), and other relevant variables (e.g. means, SDs, proportions) | 5 | Complete - In “Data sources for RHR and HRV” and “Data source for cerebral cortex SA and TH” section. |
|  | c) | If the data sources include meta-analyses of previous studies, provide the assessments of heterogeneity across these studies | NA |  |
|  | d) | For two-sample MR:  i.  Provide justification of the similarity of the genetic variant-exposure associations between the exposure and outcome samples  ii.  Provide information on the number of individuals who overlap between the exposure and outcome studies | 12 | Limited overlap may exist between the cohorts used in the exposure and outcome MR analyses. |
| 11 | **Main results** |  |  |  |
|  | a) | Report the associations between genetic variant and exposure, and between genetic variant and outcome, preferably on an interpretable scale | 7 | Complete - The associations have been reported in Table 1. |
|  | b) | Report MR estimates of the relationship between exposure and outcome, and the measures of uncertainty from the MR analysis, on an interpretable scale, such as odds ratio or relative risk per SD difference | 7 | Complete - The MR estimates have been reported in Table S6. |
|  | c) | If relevant, consider translating estimates of relative risk into absolute risk for a meaningful time period | NA |  |
|  | d) | Consider plots to visualize results (e.g. forest plot, scatterplot of associations between genetic variants and outcome versus between genetic variants and exposure) | 8 | Complete - visualize results have been reported in supplementary Fig1-14 |
| 12 | **Assessment of assumptions** |  |  |  |
|  | a) | Report the assessment of the validity of the assumptions | 7-8 | Complete – We assessed the validity using IVW, MR-Egger, weighted median as shown in the Results section |
|  | b) | Report any additional statistics (e.g., assessments of heterogeneity across genetic variants, such as *I^2^*, Q statistic or E-value) | 7-8 | Complete - The Q statistic has been reported in Table 1. |
| 13 | **Sensitivity analyses and additional analyses** |  |  |  |
|  | a) | Report any sensitivity analyses to assess the robustness of the main results to violations of the assumptions | 7-8 | Complete – We complemented IVW with MR-Egger, weighted median as sensitivity analyses. See Table S6. |
|  | b) | Report results from other sensitivity analyses or additional analyses | 7-8 | Complete – We conducted Egger intercept, Cochran’s Q test, MR-PRESSO and leave-one-out analyses. |
|  | c) | Report any assessment of direction of causal relationship (e.g., bidirectional MR) | 7-8 | Complete – We conducted steiger test. See Table S5. |
|  | d) | When relevant, report and compare with estimates from non-MR analyses | NA |  |
|  | e) | Consider additional plots to visualize results (e.g., leave-one-out analyses) | 7-8 | Complete – See supplementary Fig.7-10 |
|  | **DISCUSSION** |  |  |  |
| 14 | **Key results** | Summarize key results with reference to study objectives | 9 | Complete - Discussion paragraph 1. |
| 15 | **Limitations** | Discuss limitations of the study, taking into account the validity of the IV assumptions, other sources of potential bias, and imprecision. Discuss both direction and magnitude of any potential bias and any efforts to address them | 11 | Complete - Discussion paragraph 6. |
| 16 | **Interpretation** |  |  |  |
|  | a) | Meaning: Give a cautious overall interpretation of results in the context of their limitations and in comparison with other studies | 9 | Complete - Discussion paragraph 1. |
|  | b) | Mechanism: Discuss underlying biological mechanisms that could drive a potential causal relationship between the investigated exposure and the outcome, and whether the gene-environment equivalence assumption is reasonable. Use causal language carefully, clarifying that IV estimates may provide causal effects only under certain assumptions | 12 | Complete - In “Conclusions” section. |
|  | c) | Clinical relevance: Discuss whether the results have clinical or public policy relevance, and to what extent they inform effect sizes of possible interventions | 12 | Complete - Discussion paragraph 6. |
| 17 | **Generalizability** | Discuss the generalizability of the study results (a) to other populations, (b) across other exposure periods/timings, and (c) across other levels of exposure | 12 | Complete - Discussion paragraph 6. |
|  | **OTHER INFORMATION** |  |  |  |
| 18 | **Funding** | Describe sources of funding and the role of funders in the present study and, if applicable, sources of funding for the databases and original study or studies on which the present study is based | 14 | Complete – We have reported all sources of funding in the ”Funding” section. |
| 19 | **Data and data sharing** | Provide the data used to perform all analyses or report where and how the data can be accessed, and reference these sources in the article. Provide the statistical code needed to reproduce the results in the article, or report whether the code is publicly accessible and if so, where |  | Complete – The data used in the study can be accessed and downloaded from original studies. |
| 20 | **Conflicts of Interest** | All authors should declare all potential conflicts of interest | 14 | Complete – All authors declare that they have no conflict of interest. |

This checklist is copyrighted by the Equator Network under the Creative Commons Attribution 3.0 Unported (CC BY 3.0) license.

1. Skrivankova VW, Richmond RC, Woolf BAR, Yarmolinsky J, Davies NM, Swanson SA, et al. Strengthening the Reporting of Observational Studies in Epidemiology using Mendelian Randomization (STROBE-MR) Statement. JAMA. 2021;under review.

2. Skrivankova VW, Richmond RC, Woolf BAR, Davies NM, Swanson SA, VanderWeele TJ, et al. Strengthening the Reporting of Observational Studies in Epidemiology using Mendelian Randomisation (STROBE-MR): Explanation and Elaboration. BMJ. 2021;375:n2233.

Supplementary Fig.1 MR estimates of significant results from RHR and HRV on cortical SA and TH. (a) Leave-one-out plots from genetically predicted RHR on global SA; (b) Leave-one-out plots from genetically predicted RHR on SA of pars triangularis without global weighted; (c) Funnel plots from genetically predicted RHR on global SA; (d) Funnel plots from genetically predicted RHR on SA of pars triangularis without global weighted; (e) Leave-one-out plots from genetically predicted RHR on TH of banks of the superior temporal sulcus with global weighted; (f) Leave-one-out plots from genetically predicted RHR on TH of paracentral with global weighted; (g) Funnel plots from genetically predicted RHR on TH of banks of the superior temporal sulcus with global weighted; (h) Funnel plots from genetically predicted RHR on TH of paracentral with global weighted.


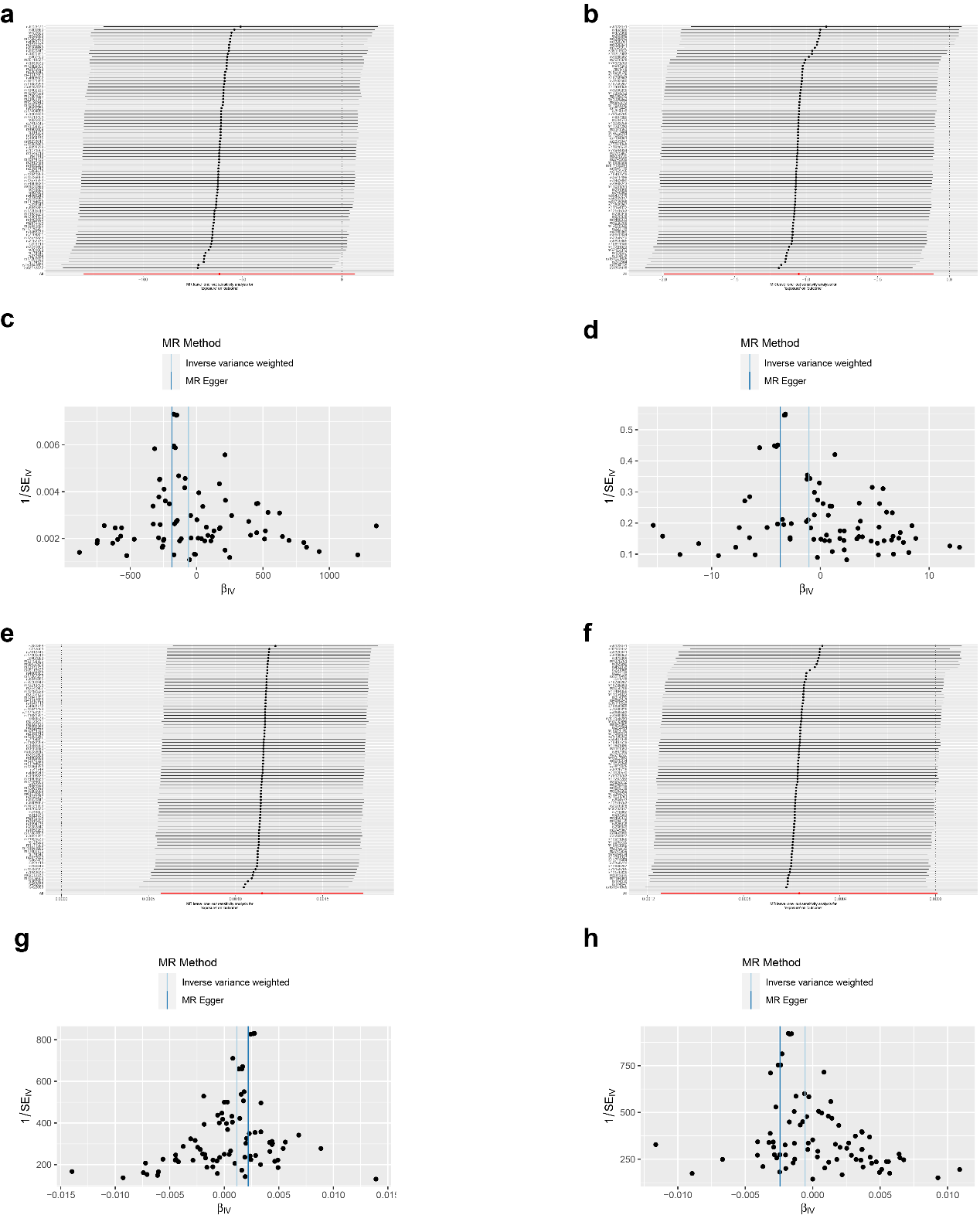


Supplementary Fig.2 MR estimates of significant results from RHR and HRV on cortical SA and TH after removal of risk SNPs in phenoscanner. (a) Scatter plots from genetically predicted RHR on global SA; (b) Scatter plots from genetically predicted RHR on SA of pars triangularis without global weighted; (c) Funnel plots from genetically predicted RHR on global SA; (d) Funnel plots from genetically predicted RHR on SA of pars triangularis without global weighted; (e) Leave-one-out plots from genetically predicted RHR on global SA; (f) Leave-one-out plots from genetically predicted RHR on SA of pars triangularis without global weighted; (g) Scatter plots from genetically predicted RHR on TH of banks of the superior temporal sulcus with global weighted; (h) Scatter plots from genetically predicted RHR on TH of paracentral with global weighted; (i) Funnel plots from genetically predicted RHR on TH of banks of the superior temporal sulcus with global weighted; (j) Funnel plots from genetically predicted RHR on TH of paracentral with global weighted; (k) Leave-one-out plots from genetically predicted RHR on TH of banks of the superior temporal sulcus with global weighted; (l) Leave-one-out plots from genetically predicted RHR on TH of paracentral with global weighted.


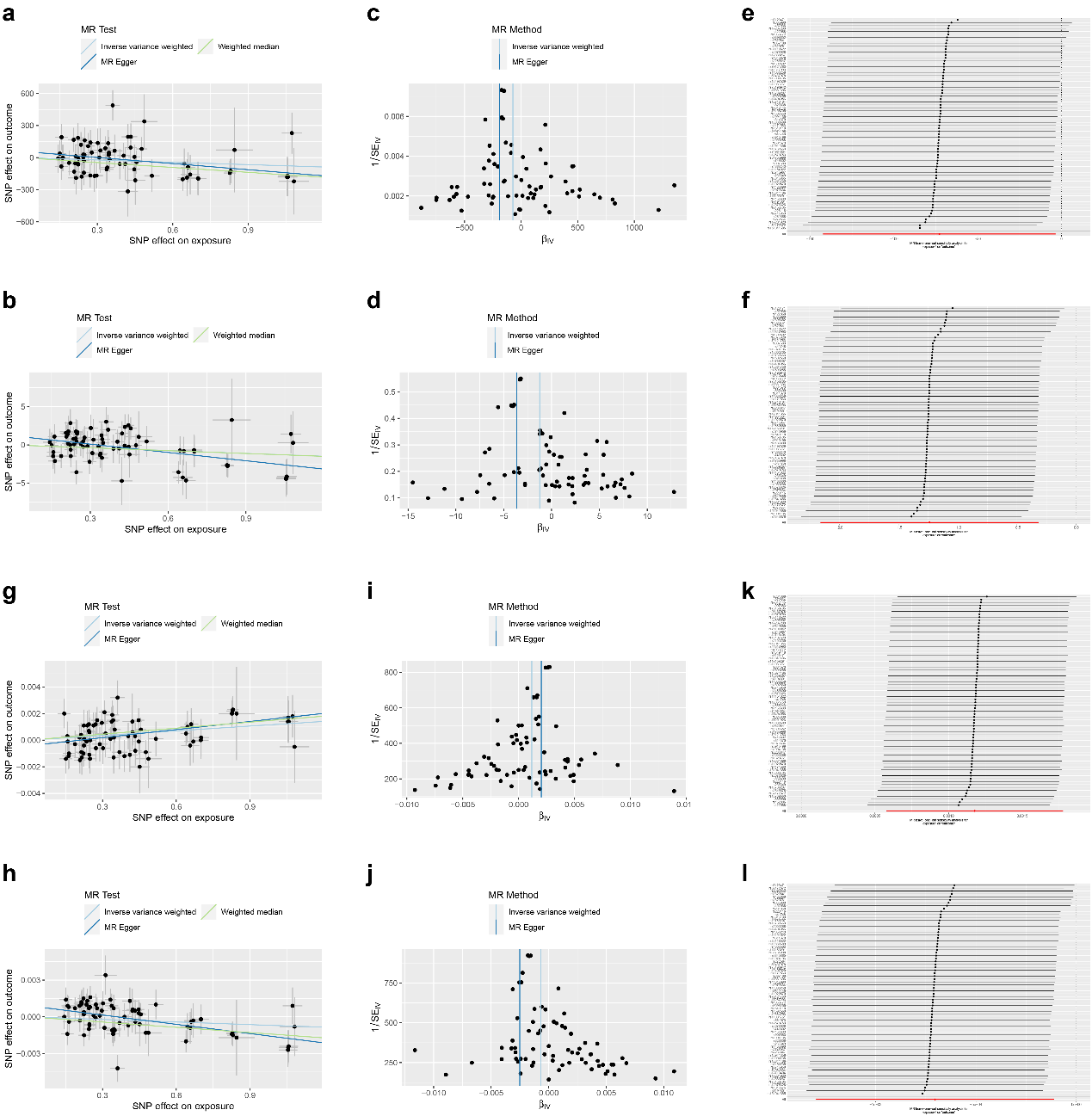


Supplementary Fig.3 Scatter plots of nominal significant estimates from genetically predicted RHR with global weighted on the cortical structure. (a) TH of the caudal anterior cingulate; (b) TH of the fusiform; (c) TH of theinferiorparietal; (d)TH of the lateral occipital; (e) TH of the paracentral ;(f)TH of the pars opercularis; (g) TH of the pars triangularis; (h)TH of the postcentral; (i)TH of the posterior cingulate; (j) TH of the precentral; (k)TH of the superior frontal; (l)TH of the temporal pole; (m) SA of the entorhinal; (n) SA of the fusiform; (o) SA of the inferior temporal; (p) SA of the lingual; (q) SA of the paracentral; (r) SA of the pars triangularis; (s) SA of the precentral; (t) SA of the rostral middle frontal; (u) SA of the superior temporal; (v) SA of the temporal pole.


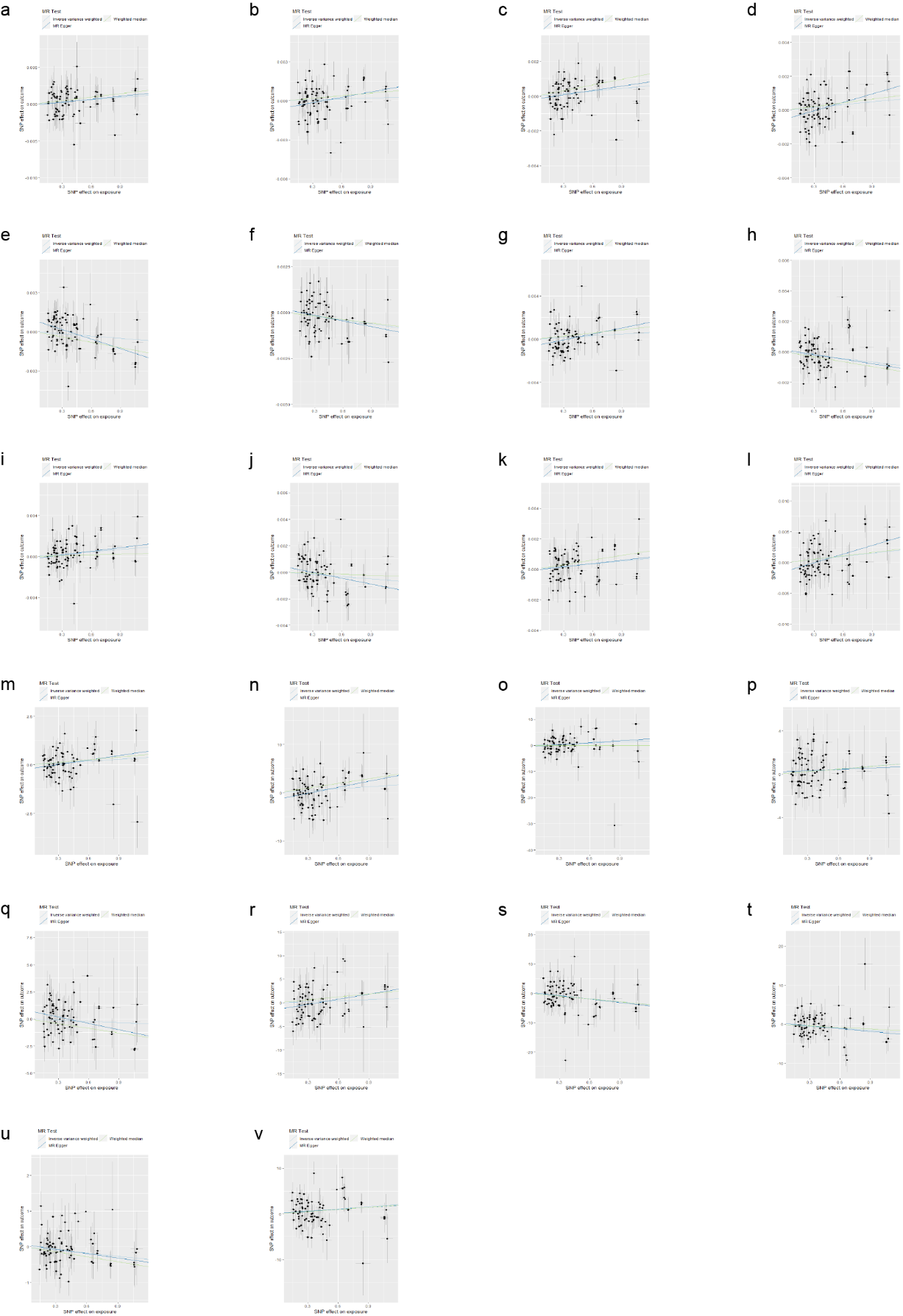


Supplementary Fig.4 Scatter plots of nominal significant estimates from genetically predicted RHR without global weighted on the cortical structure. (a) SA of the caudal anterior cingulate; (b) SA of the insula; (c) SA of the isthmuscingulate; (d) SA of the middle temporal; (e) SA of the pars opercularis; (f) SA of the pars orbitalis; (g) SA of the posterior cingulate; (h) SA of the rostral middle frontal; (i) SA of the superior temporal; (j) SA of the supramarginal; (k) SA of the temporal pole; (l) SA of the transverse temporal; (m) TH of the banks of the superior temporal sulcus; (n) TH of the lateral occipital; (o) TH of the paracentral; (p) TH of the pars triangularis; (q) TH of the temporal pole.


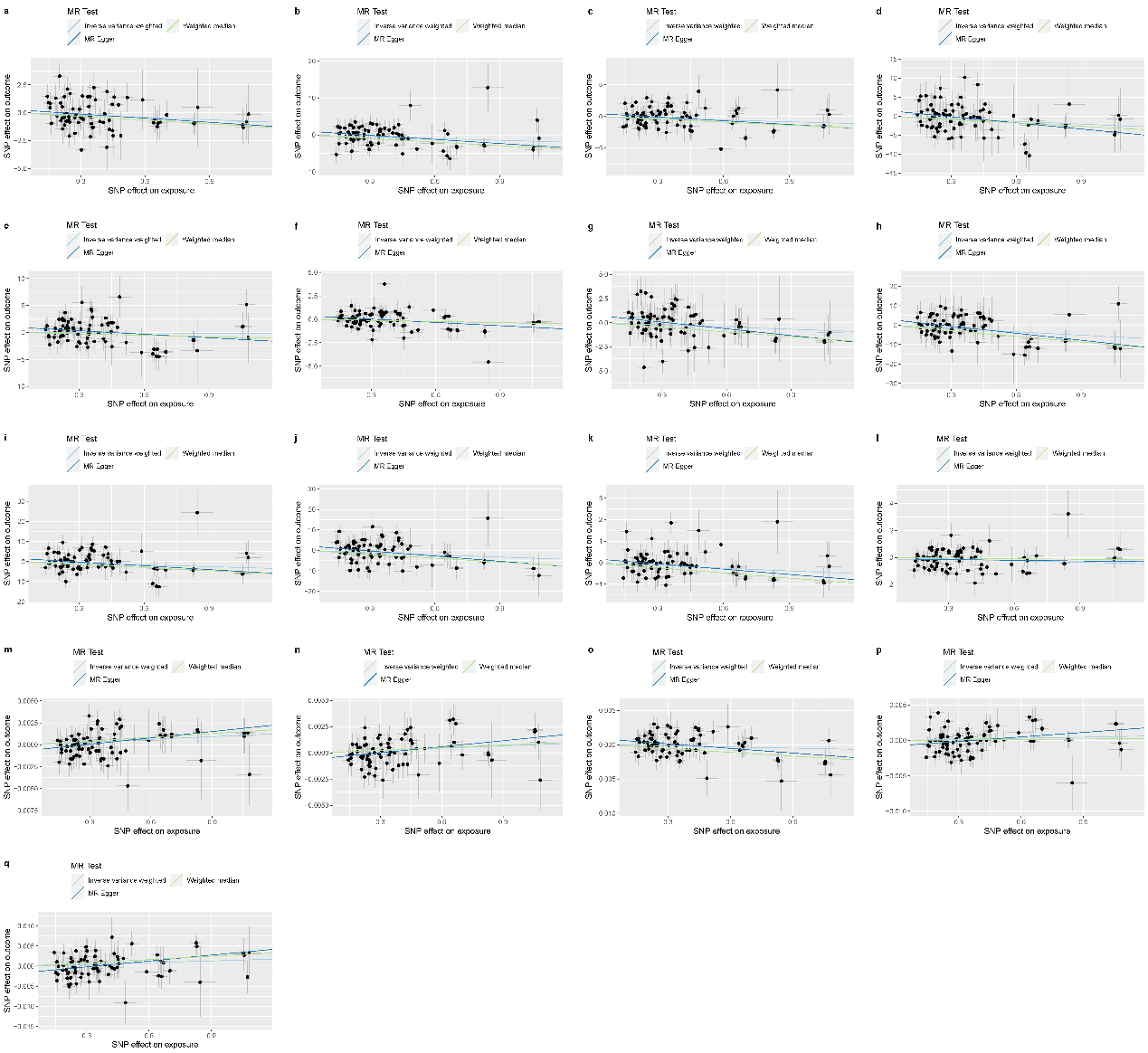


Supplementary Fig.5 Scatter plots of nominal significant estimates from genetically predicted HRV with global weighted on the cortical structure. (a) SA of the temporal pole; (b)TH of the caudal anterior cingulate; (c) TH of the superior temporal; (d) TH of the supramarginal.


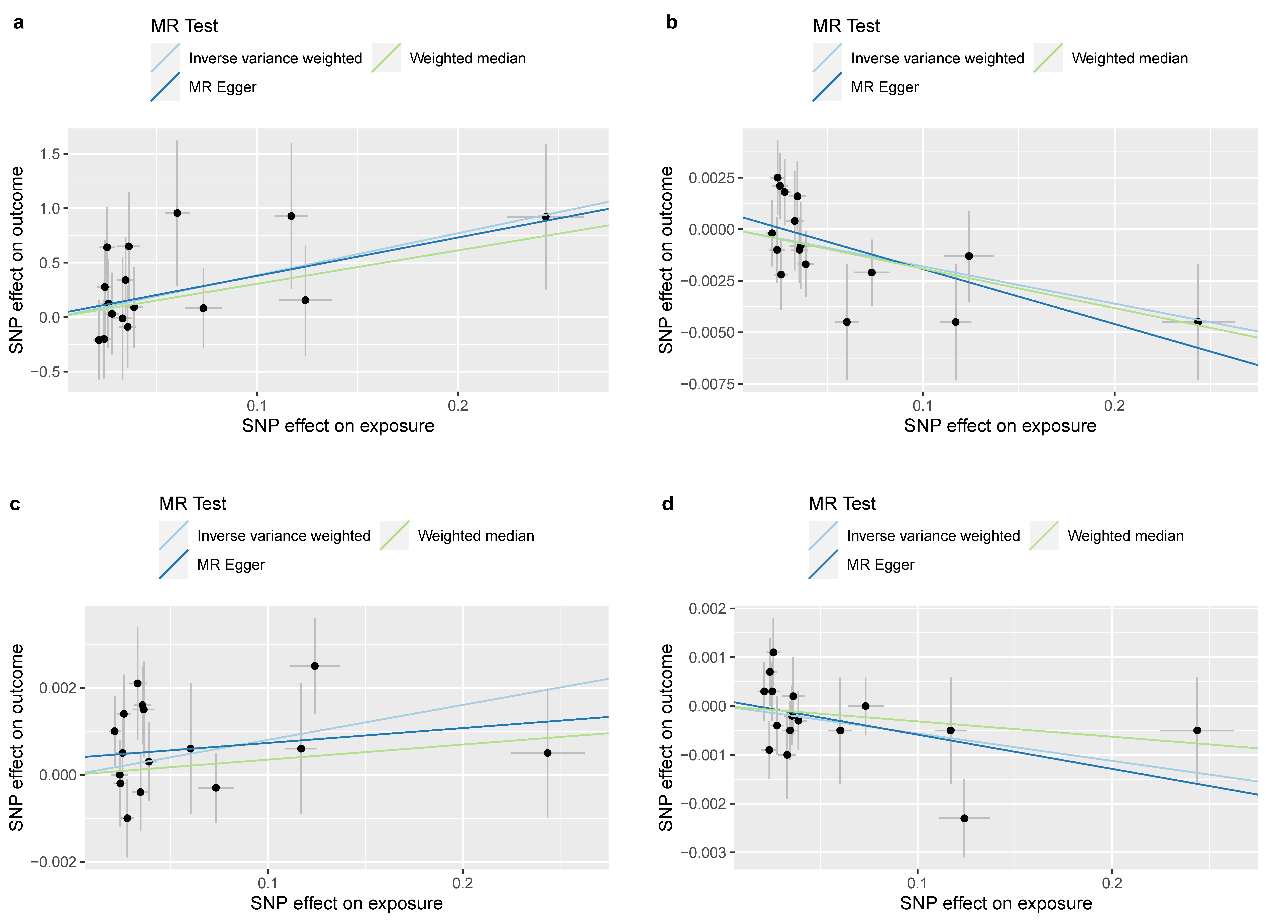


Supplementary Fig.6 Scatter plots of nominal significant estimates from genetically predicted HRV without global weighted on the cortical structure. (a) SA of the lingual; (b) SA of the temporal pole; (c) TH of the caudal anterior cingulate; (d) TH of the posterior cingulate; (e) TH of the superiorfrontal.


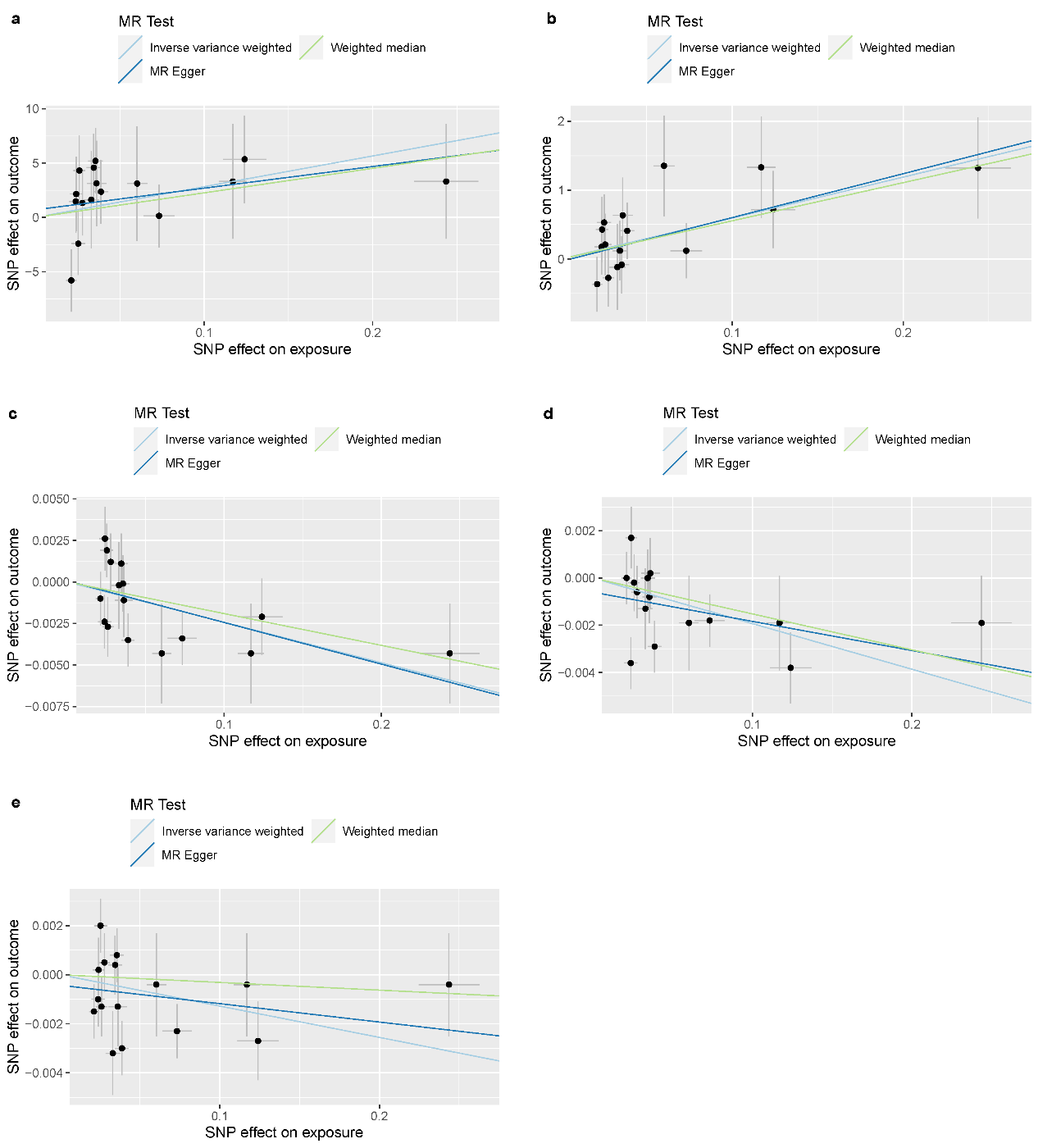


Supplementary Fig.7 Leave-one-out plots of nominal significant estimates from genetically predicted RHR with global weighted on the cortical structure. (a) TH of the caudal anterior cingulate; (b) TH of the fusiform; (c) TH of theinferiorparietal; (d)TH of the lateral occipital; (e) TH of the paracentral ;(f)TH of the pars opercularis; (g) TH of the pars triangularis; (h)TH of the postcentral; (i)TH of the posterior cingulate; (j) TH of the precentral; (k)TH of the superior frontal; (l)TH of the temporal pole; (m) SA of the entorhinal; (n) SA of the fusiform; (o) SA of the inferior temporal; (p) SA of the lingual; (q) SA of the paracentral; (r) SA of the pars triangularis; (s) SA of the precentral; (t) SA of the rostral middle frontal; (u) SA of the superior temporal; (v) SA of the temporal pole.


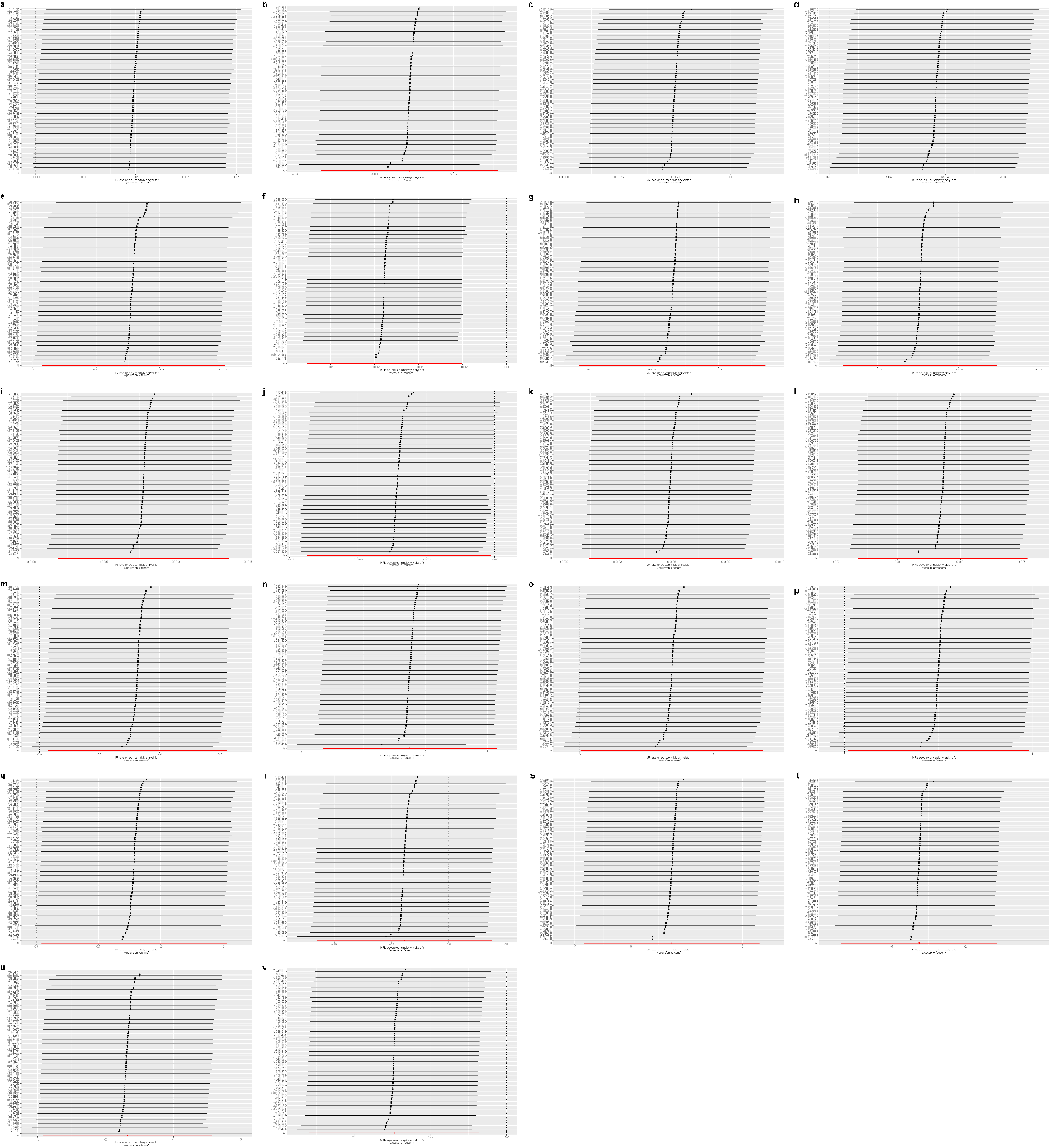


Supplementary Fig.8 Leave-one-out plots of nominal significant estimates from genetically predicted RHR without global weighted on the cortical structure. (a) SA of the caudal anterior cingulate; (b) SA of the insula; (c) SA of the isthmuscingulate; (d) SA of the middle temporal; (e) SA of the pars opercularis; (f) SA of the pars orbitalis; (g) SA of the posterior cingulate; (h) SA of the rostral middle frontal; (i) SA of the superior temporal; (j) SA of the supramarginal; (k) SA of the temporal pole; (l) SA of the transverse temporal; (m) TH of the banks of the superior temporal sulcus; (n) TH of the lateral occipital; (o) TH of the paracentral; (p) TH of the pars triangularis; (q) TH of the temporal pole.


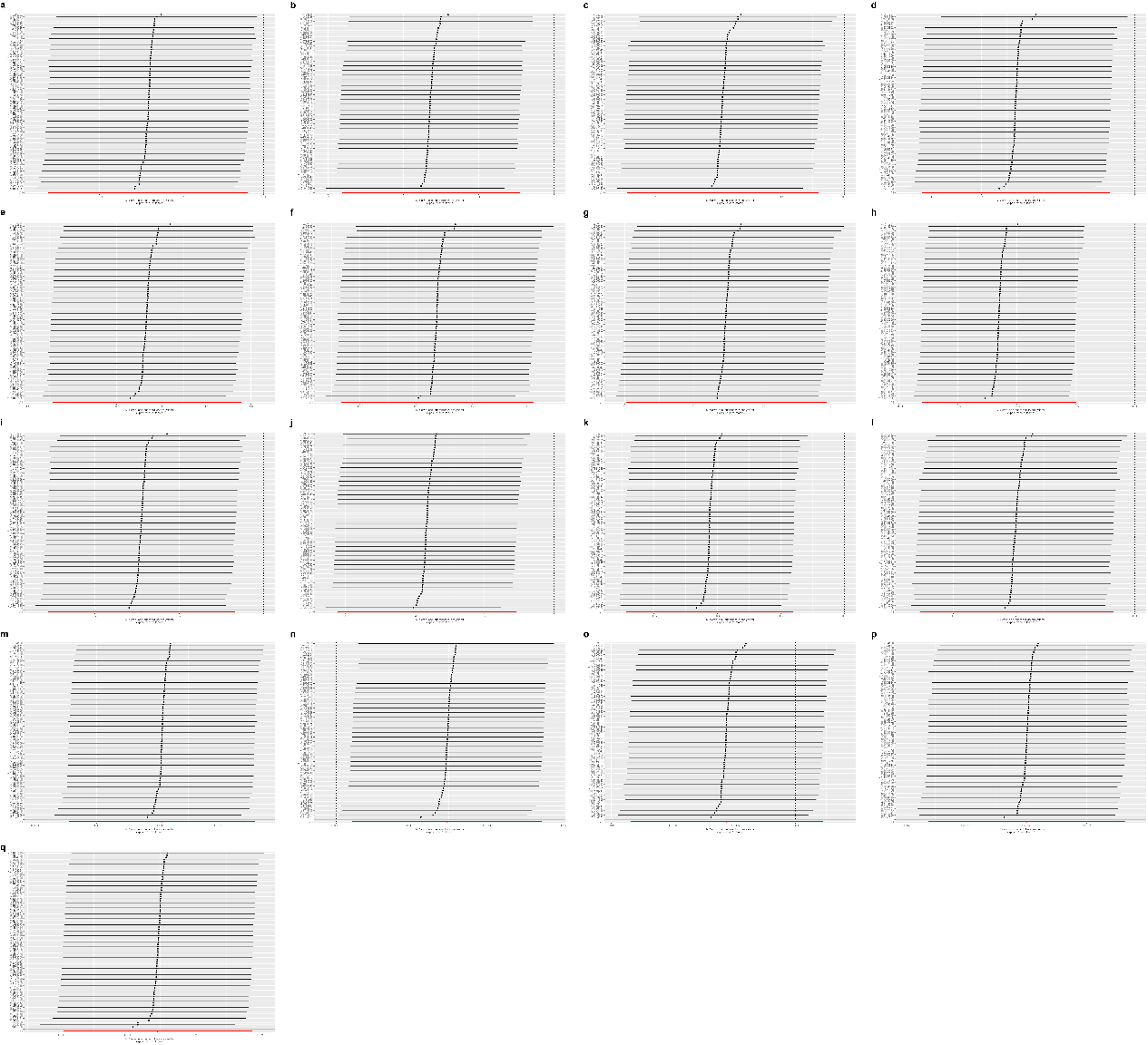


Supplementary Fig.9 Leave-one-out plots of nominal significant estimates from genetically predicted HRV with global weighted on the cortical structure. (a) SA of the temporal pole; (b)TH of the caudal anterior cingulate; (c) TH of the superior temporal; (d) TH of the supramarginal.


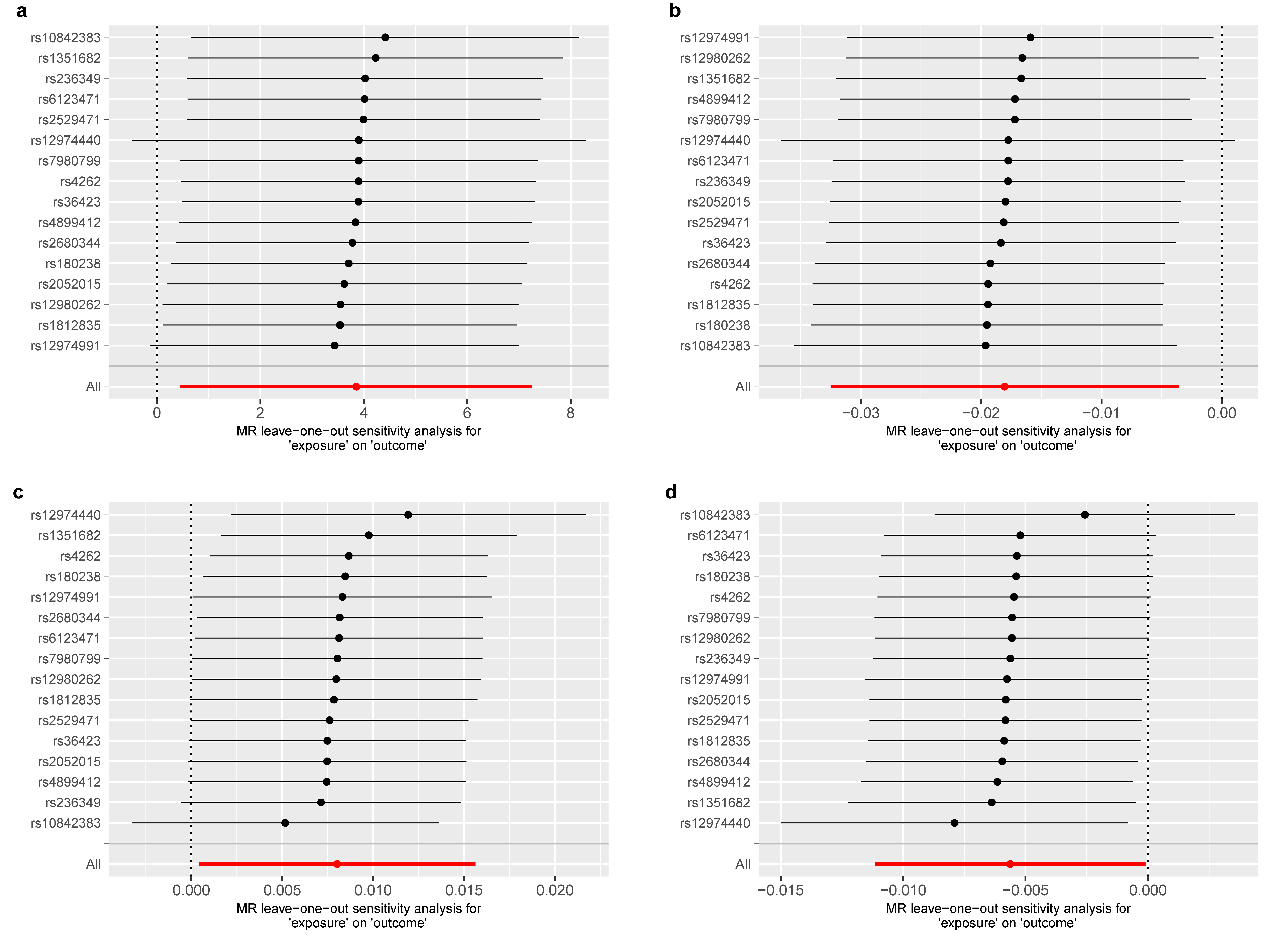


Supplementary Fig.10 Leave-one-out plots of nominal significant estimates from genetically predicted HRV without global weighted on the cortical structure. (a) SA of the lingual; (b) SA of the temporal pole; (c) TH of the caudal anterior cingulate; (d) TH of the posterior cingulate; (e) TH of the superior frontal.


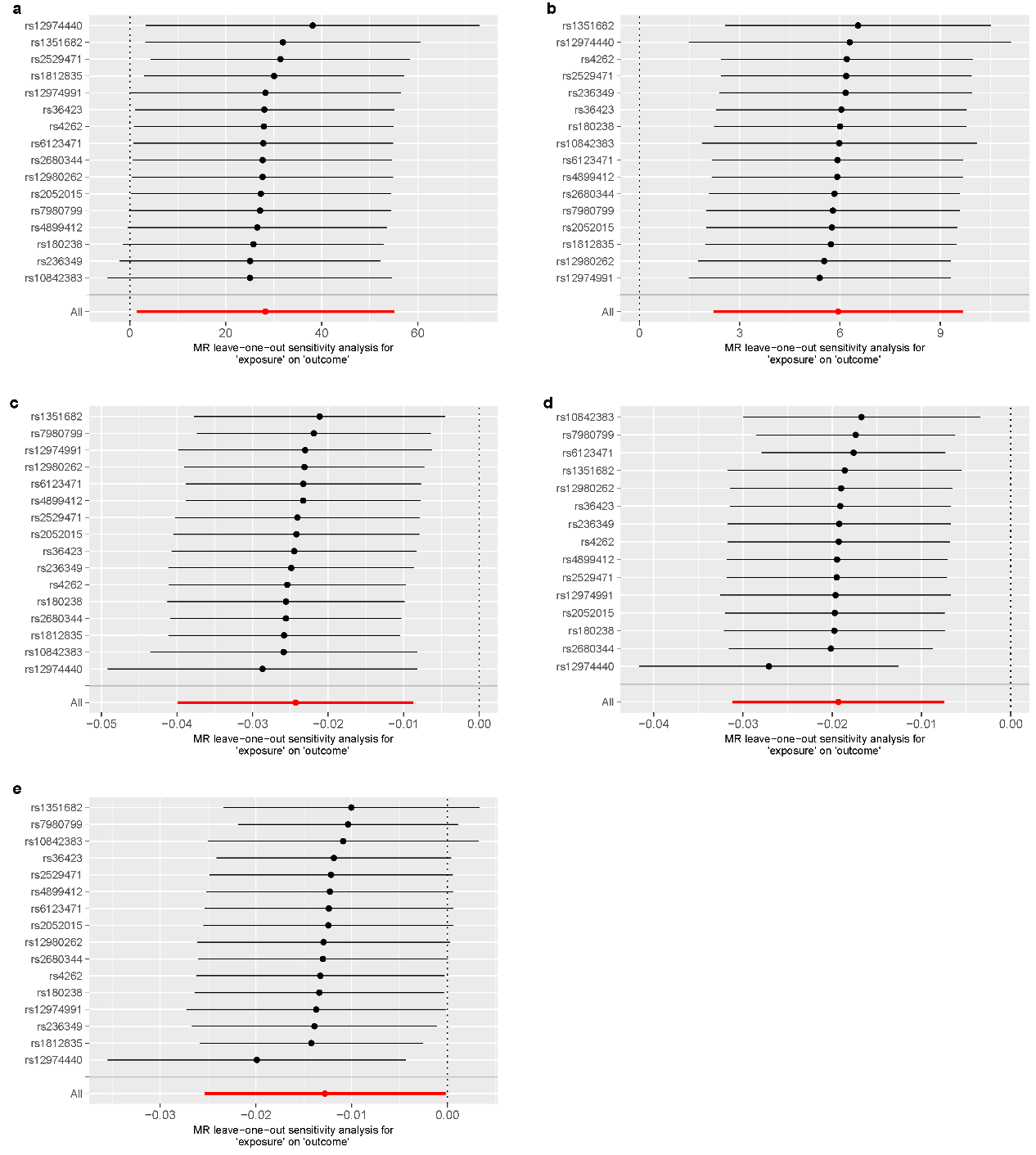


Supplementary Fig.11 Funnel plots of nominal significant estimates from genetically predicted RHR with global weighted on the cortical structure. (a) TH of the caudal anterior cingulate; (b) TH of the fusiform; (c) TH of theinferiorparietal; (d)TH of the lateral occipital; (e) TH of the paracentral ;(f)TH of the pars opercularis; (g) TH of the pars triangularis; (h)TH of the postcentral; (i)TH of the posterior cingulate; (j) TH of the precentral; (k)TH of the superior frontal; (l)TH of the temporal pole; (m) SA of the entorhinal; (n) SA of the fusiform; (o) SA of the inferior temporal; (p) SA of the lingual; (q) SA of the paracentral; (r) SA of the pars triangularis; (s) SA of the precentral; (t) SA of the rostral middle frontal; (u) SA of the superior temporal; (v) SA of the temporal pole.


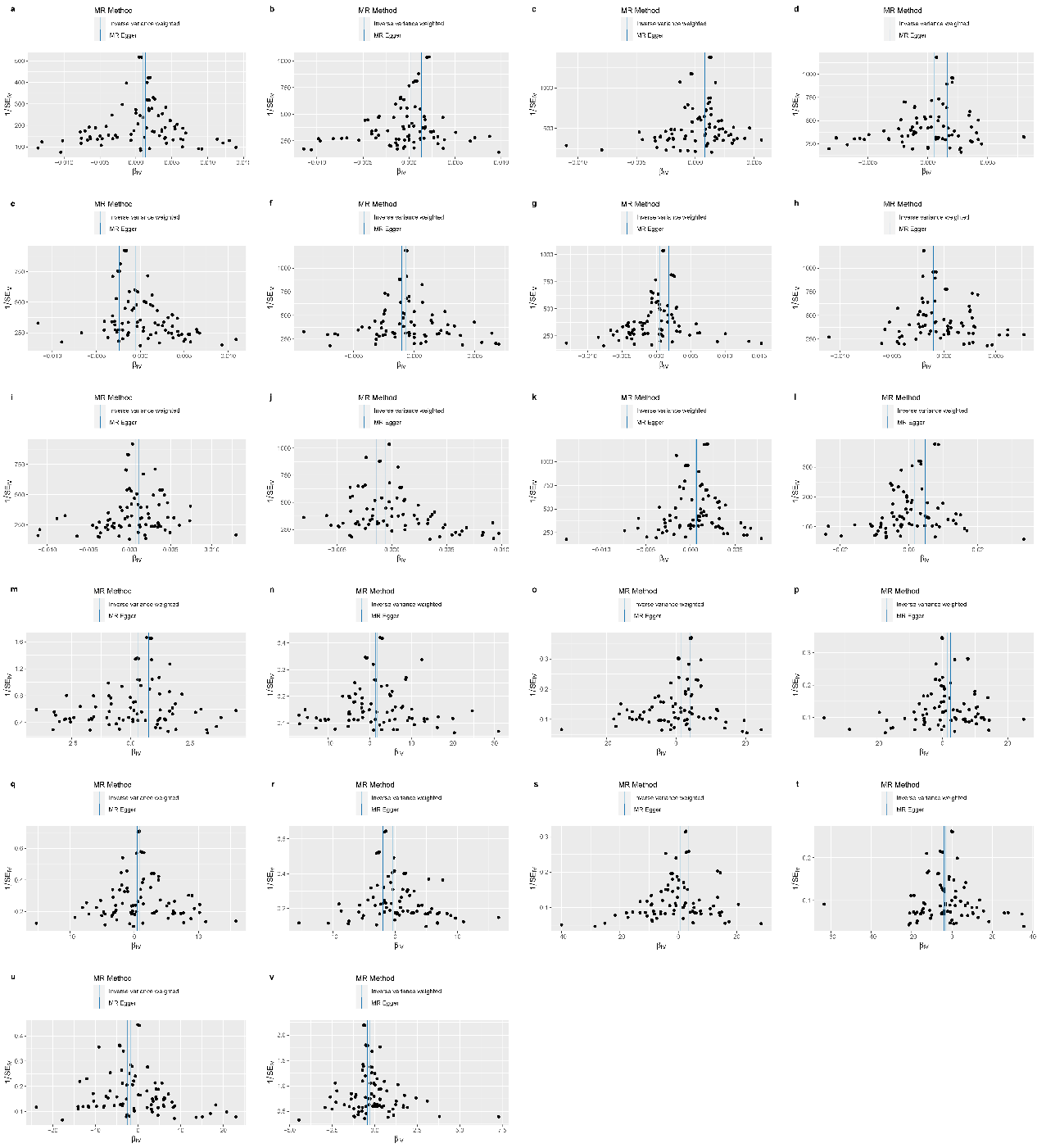


Supplementary Fig.12 Funnel plots of nominal significant estimates from genetically predicted RHR without global weighted on the cortical structure. (a) SA of the caudal anterior cingulate; (b) SA of the insula; (c) SA of the isthmuscingulate; (d) SA of the middle temporal; (e) SA of the pars opercularis; (f) SA of the pars orbitalis; (g) SA of the posterior cingulate; (h) SA of the rostral middle frontal; (i) SA of the superior temporal; (j) SA of the supramarginal; (k) SA of the temporal pole; (l) SA of the transverse temporal; (m) TH of the banks of the superior temporal sulcus; (n) TH of the lateral occipital; (o) TH of the paracentral; (p) TH of the pars triangularis; (q) TH of the temporal pole.


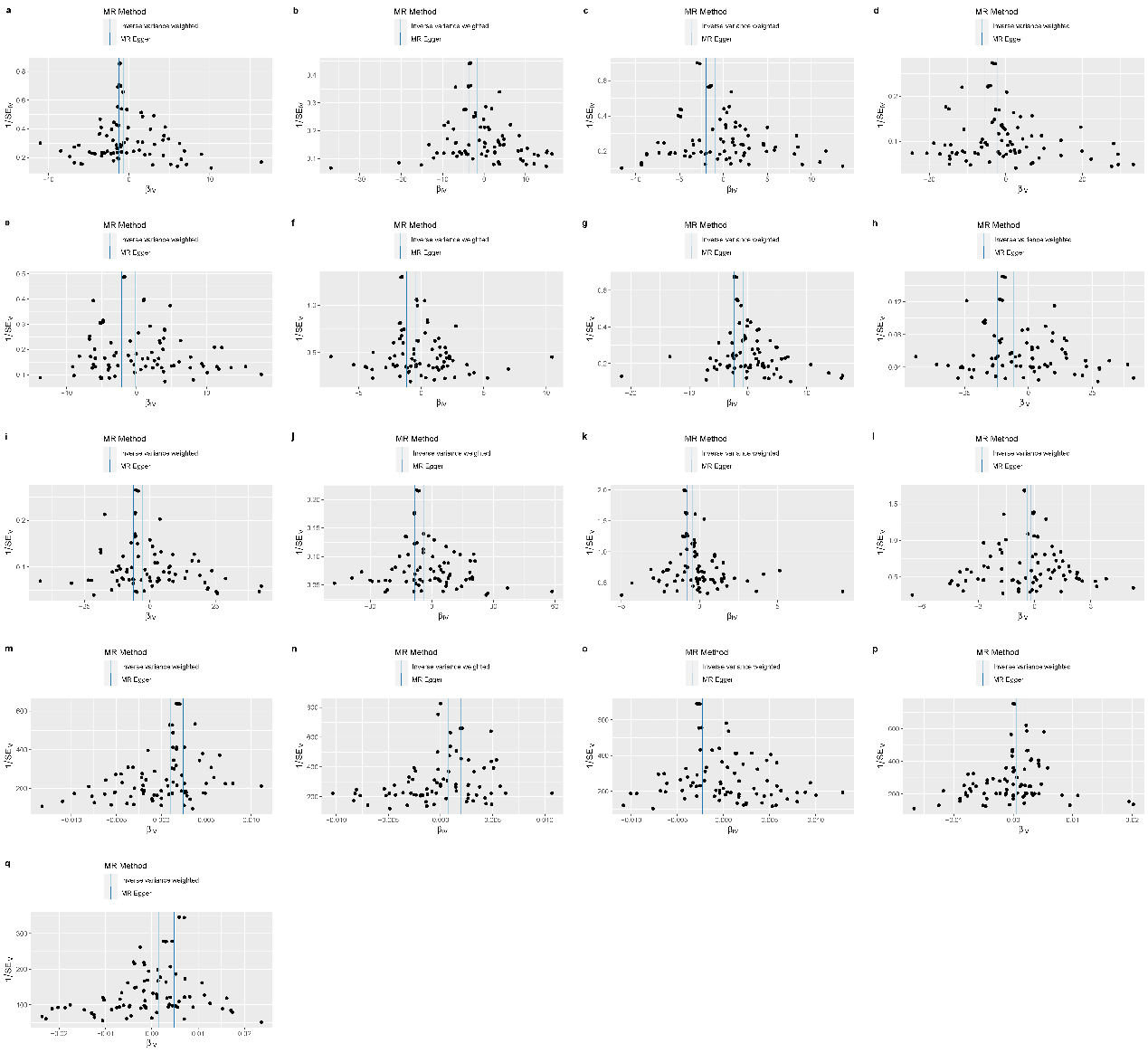


Supplementary Fig.13 Funnel plots of nominal significant estimates from genetically predicted HRV with global weighted on the cortical structure. (a) SA of the temporal pole; (b) TH of the caudal anterior cingulate; (c) TH of the superior temporal; (d) TH of the supramarginal.


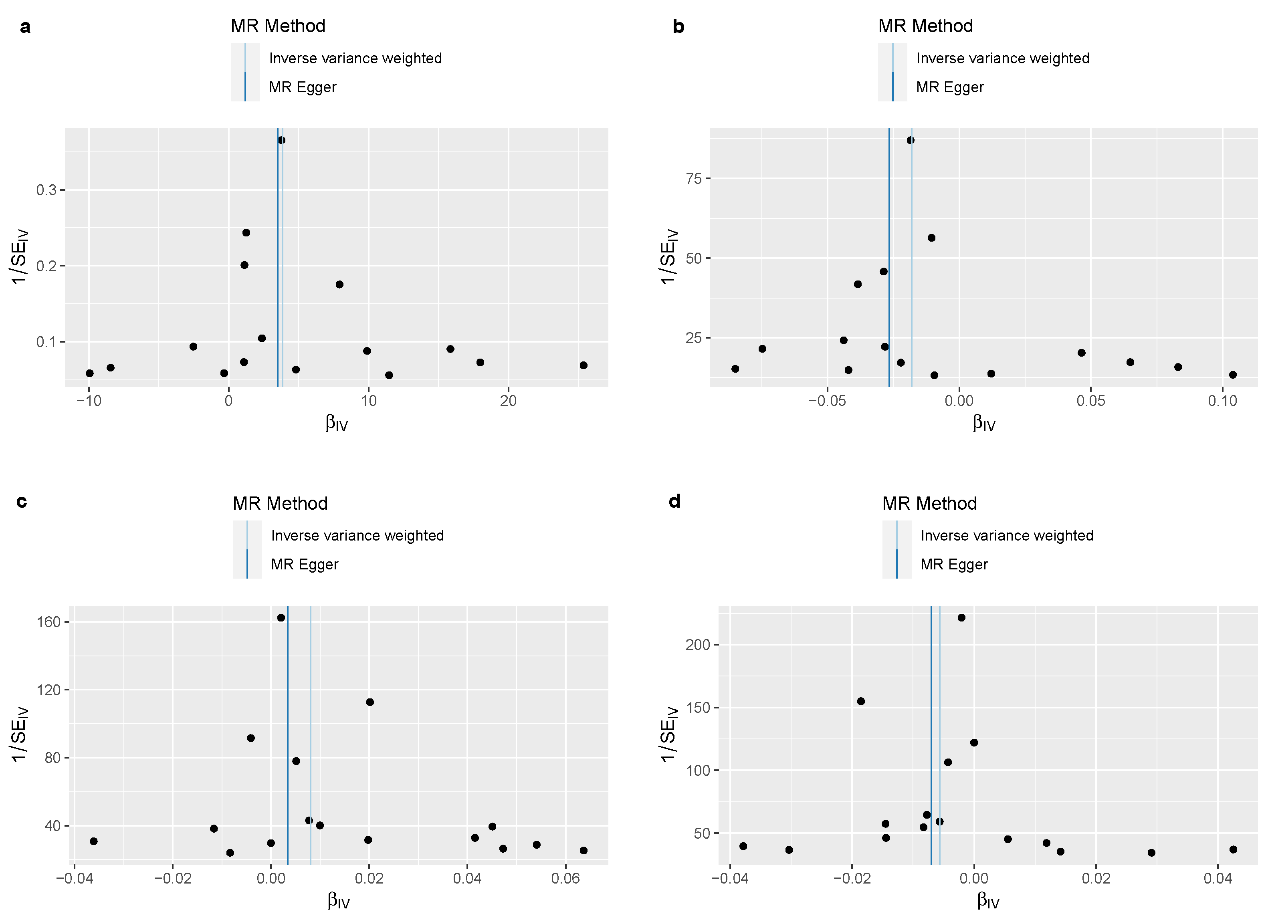


Supplementary Fig.14 Funnel plots of nominal significant estimates from genetically predicted HRV without global weighted on the cortical structure. (a) SA of the lingual; (b) SA of the temporal pole; (c) TH of the caudal anterior cingulate; (d) TH of the posterior cingulate; (e) TH of the superior frontal.


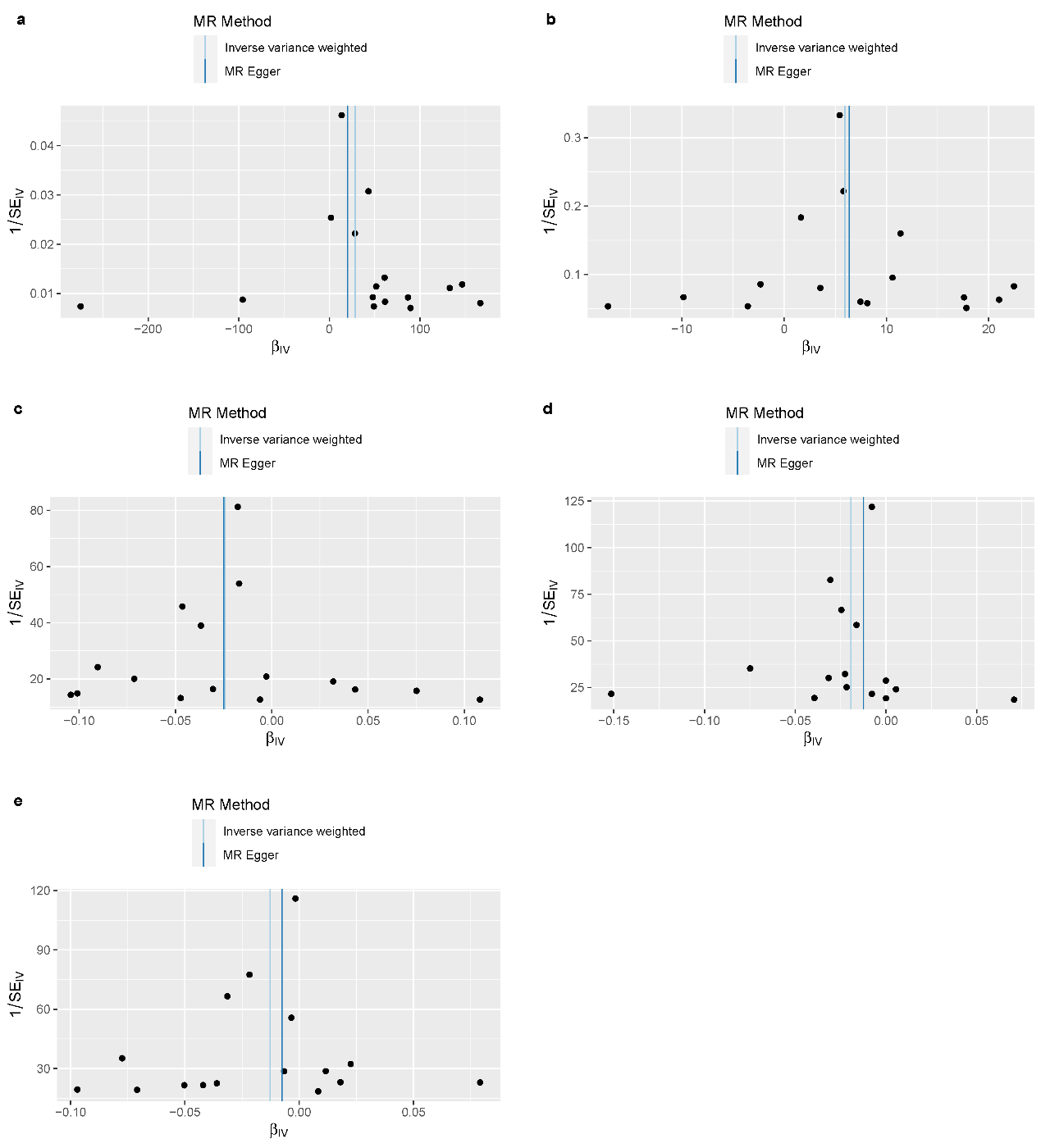

Supplement: Supplementary_Materials_bhad536 [file supplementary_materials_bhad536.docx]
